# Mortality risk from United States coal electricity generation

**DOI:** 10.1126/science.adf4915

**Published:** 2023-11-23

**Authors:** Lucas Henneman, Christine Choirat, Irene Dedoussi, Francesca Dominici, Jessica Roberts, Corwin Zigler

**Affiliations:** 1 Department of Civil, Environmental, and Infrastructure Engineering, George Mason University Volgenau School of Engineering, Fairfax, VA, USA.; 2 Department of Biostatistics, Harvard T.H. Chan School of Public Health, Harvard Data Science Initiative, Harvard University, Boston, MA, USA.; 3 Institute of Global Health, Faculty of Medicine, University of Geneva, Geneva, Switzerland.; 4 Section Aircraft Noise and Climate Effects, Faculty of Aerospace Engineering, Delft University of Technology, Delft, Netherlands.; 5 School of Interactive Computing, Georgia Institute of Technology, Atlanta, GA, USA.; 6 Department of Statistics and Data Sciences, University of Texas, Austin, TX, USA.

## Abstract

Policy-makers seeking to limit the impact of coal electricity-generating units (EGUs, also known as power plants) on air quality and climate justify regulations by quantifying the health burden attributable to exposure from these sources. We defined “coal PM_2.5_” as fine particulate matter associated with coal EGU sulfur dioxide emissions and estimated annual exposure to coal PM_2.5_ from 480 EGUs in the US. We estimated the number of deaths attributable to coal PM_2.5_ from 1999 to 2020 using individual-level Medicare death records representing 650 million person-years. Exposure to coal PM_2.5_ was associated with 2.1 times greater mortality risk than exposure to PM_2.5_ from all sources. A total of 460,000 deaths were attributable to coal PM_2.5_, representing 25% of all PM_2.5_-related Medicare deaths before 2009 and 7% after 2012. Here, we quantify and visualize the contribution of individual EGUs to mortality.

## Editor’s summary

The success of measures to mitigate environmental damage can be hard to assess. The advent of new modeling tools brings us closer to estimates that are reproducible and do not need expensive and time-consuming computing. Henneman *et al*. found that coal-burning power stations emit fine particulates (PM_2.5_) containing sulfur dioxide that are associated with higher mortality than other types of PM_2.5_ (see the Perspective by Mendelsohn and Min Kim). Using a reduced-form atmospheric model combined with historical Medicare data from the US, the authors identified the coal-burning power plants associated with the greatest mortality and estimated the effect that closure or scrubber installation has had on reducing it. This type of approach can provide a rapid indication of the effectiveness of environmental protection measures to inform ongoing policy decisions. —Caroline Ash

Air pollution exposure is associated with adverse health effects and increased risk of death (1-4). Coal electricity-generating units (EGUs), or power plants, are a major contributor to poor air quality (5-7). Coal, historically a relatively inexpensive fuel, is burned to provide electricity worldwide even as the US and other nations continue to debate whether it should remain a part of the energy portfolio amid public health and climate concerns. Global coal use for electricity generation is projected to increase (8), and ongoing instability has pushed European nations to increase coal use (9, 10). Although coal EGU air pollution emissions have declined in the US in recent decades (11), defining the health burden posed by coal EGUs and the benefits of actions that have reduced EGU emissions remains paramount to informing public health, climate, and energy policies in the US (12) and worldwide.

Previous studies that quantified the mortality burden from coal EGUs in the US (13-18) relied on estimated concentration response functions (CRFs), which assume that fine particulate matter (PM_2.5_) from coal emissions has the same toxicity as PM_2.5_ from all sources. However, evidence indicates (19-25) that exposure to sulfur, sulfates, or PM_2.5_ from coal emissions may be associated with higher relative morbidity or mortality risk than that to other PM_2.5_ constituents or PM_2.5_ from other sources per unit concentration, although uncertainty remains (26, 27). The limited regional (19-22) and temporal (23-25) scope of previous studies, along with the lack of availability of coal-specific exposure estimates, has hindered the adoption of coal-specific PM_2.5_ CRFs in mortality burden calculations, likely leading to underestimates of the mortality burden associated with coal EGUs. In addition, previous studies lack targeted evidence regarding which coal EGUs are most responsible for increased mortality risk, and this information is needed to inform policies.

To estimate the number of deaths associated with exposure to coal PM_2.5_ from EGUs, we conducted a national-scale study of individuallevel health records covering >650 million person-years in the US Medicare population (≥65 years of age) from 1999 to 2016 (unless otherwise noted, populations throughout this study refer specifically to the Medicare population) (28). We defined “coal PM_2.5_” as PM_2.5_ from coal EGU SO_2_ emissions. We estimated coal PM_2.5_ using the HYSPLIT with Average Dispersion (HyADS) model, which accounts for date-specific atmospheric transport of PM_2.5_ to characterize exposure to PM_2.5_ from individual EGUs (29-32). We used HyADS, a reduced complexity model, to estimate 22 years of exposure to coal PM_2.5_ (from 1999 to 2020) from each of 480 US EGUs. These calculations would have required multiple orders of magnitude more computation time using a typical full-scale chemical transport model.

Our study offers the following contributions. First, we estimated and compared mortality risk associated with exposure to coal PM_2.5_ versus total PM_2.5_ from all sources, showing that previous analyses underestimated the mortality burden from coal EGUs in the US. Second, we calculated the number of deaths linked to each of the 480 coal EGUs, ranking each with respect to its contribution to the mortality burden and tracking its contribution to the overall mortality burden over time amid implementation of emissions controls and retirements. Third, we documented the spatial distribution of the mortality burden across the US.

## Results

### Changes in exposure to coal PM_2.5_ over time

**Fig. 1.**
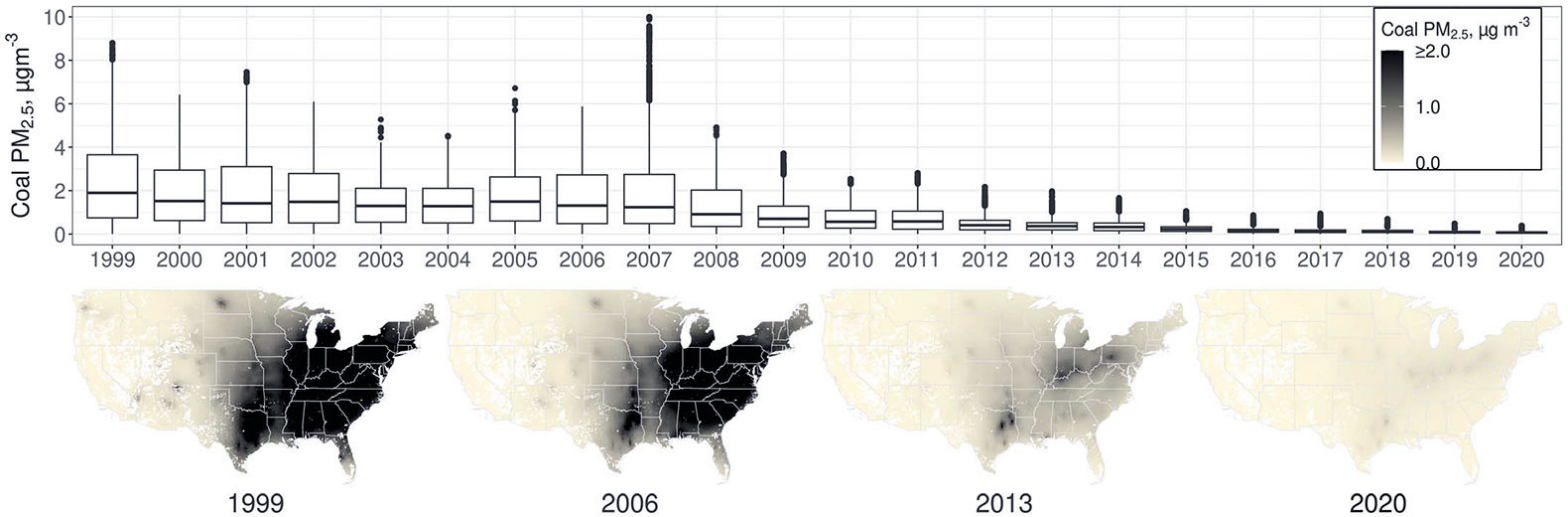
ZIP code–level coal PM_2.5_ over time. Box plots (median, first, and third quartiles are shown as horizonal lines and outliers as dots) summarize the distribution of ZIP code levels of coal PM_2.5_. Map areas shown in white do not have ZIP codes. Plots were produced in R using ggplot2; spatial information comes from the USAboundaries package.

By averaging ZIP (postal) code levels of coal PM_2.5_ across the conterminous US, we found that the annual average coal PM_2.5_ declined from 2.34 μg m^−3^ (range, 0.01 to 8.80) in 1999 to 0.07 μg m^−3^ (range, 0.00 to 0.39) in 2020 (Fig. 1). Coal PM_2.5_ was elevated in the eastern US relative to the western US, with annual average concentrations exceeding 4 μg m^−3^ in multiple ZIP codes in all years from 1999 to 2008. Coal PM_2.5_ exposure is a combination of emissions from nearby and distant EGUs (figs. S1 and S2).

### Coal PM_2.5_ CRF

The Medicare dataset contains records of 32.5 million deaths from 1999 to 2016 (table S1), with the annual number of deaths increasing and death rates decreasing across the study period (fig. S3). We found that a 1 μg m^−3^ increase in annual average coal PM_2.5_ was associated with a 1.12% increase in all-cause mortality [relative risk (RR): 1.0125; 95% confidence interval (CI): 1.0113 to 1.0137]). This risk is ~2.1 times greater than the RR associated with exposure to PM_2.5_ from any source (1.0060 per μg m^−3^; 95% CI: 1.0053 to 1.0067), which was estimated by Wu *et al.* in 2020 in the same Medicare cohort using an analogous statistical model (4).

### Number of excess deaths attributable to coal PM_2.5_

**Fig. 2.**
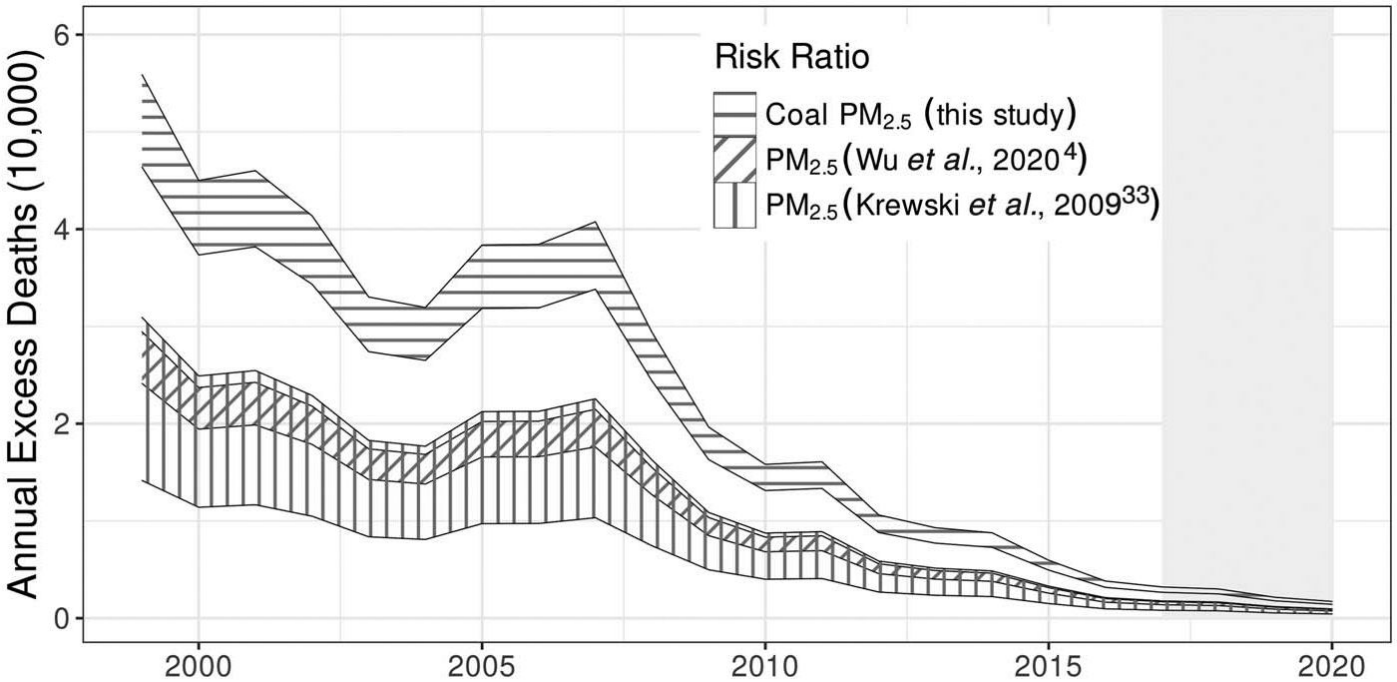
Annual number of excess deaths attributable to coal PM_2.5_, estimated using the RR for coal PM_2.5_ from this study and RRs for total PM_2.5_ from the literature. All excess deaths are estimated relative to zero coal PM_2.5_. The area filled by horizontal hashing indicates deaths estimated using RRs derived from this study (bounds represent 95% CI). Areas filled by vertical and diagonal hashing correspond to deaths estimated using RRs for total ambient PM_2.5_ exposure from the literature (4, 33). The gray shaded region from 2017 to 2020 represents years for which ZIP code–specific baseline death rates were assumed from the 2014 to 2016 average. This figure was produced in R using ggplot2.

For each year from 1999 to 2020, we estimated the excess number of deaths attributable to coal PM_2.5_ relative to what would have occurred assuming zero SO_2_ emissions from coal EGUs (i.e., coal PM_2.5_ = 0). Summing over the study period, we estimated that 460,000 (95% CI: 420,000 to 500,000) deaths would have been avoided if all coal EGU SO_2_ emissions were eliminated (Fig. 2 and table S2). Annual excess deaths attributable to coal PM_2.5_ were highest between 1999 and 2007, averaging more than 43,000 deaths per year for a total of 390,000 (95% CI: 360,000 to 430,000). After 2007, annual excess deaths declined substantially, reaching 1600 (95% CI: 1400 to 1700) in 2020. The total number of deaths in the Medicare population for the period 1999 to 2020 was 38.6 million (we projected annual deaths in each ZIP code for the period 2017 to 2020 as the average from 2014 to 2016; fig. S3). Therefore, Medicare deaths associated with coal PM_2.5_ exposure represent 1.2% (95% CI: 1.1 to 1.3%) of all Medicare deaths. Changes in baseline mortality rates had a much smaller influence than changes in coal PM_2.5_ on the variability in annual deaths associated with coal PM_2.5_ since 1999 (figs. S4 and S5).

The estimated RR for coal PM_2.5_ from the statistical model was higher than the previously estimated RRs for total PM_2.5_ exposure that are often used for risk assessments, implying that the number of excess deaths attributable to coal EGUs was underestimated in prior studies (13-18). For example, by combining coal PM_2.5_ exposure with two RRs for total PM_2.5_ previously used in risk assessments, 1.0060 per 1 μg m^−3^ (95% CI: 1.0053 to 1.0067) estimated for the Medicare population (4) and 1.6% per 10 μg m^−3^ (95% CI: 1.4 to 1.8) estimated for the general population (33), we estimated 240,000 (95% CI: 220,000 to 260,000) and 200,000 (95% CI: 130,000 to 280,000) excess deaths from coal EGUs, respectively (Fig. 2).

We compared mortality from coal PM_2.5_ estimated in the main analysis with an aggregate health burden associated with total PM_2.5_ from all sources. Using the RR reported by Wu et al.(4) for the Medicare population and the same annual PM_2.5_ exposure used in that analysis, we calculated 2,000,000 excess deaths due to ambient PM_2.5_ from 2000 to 2016 relative to a PM_2.5_ concentration of 0 (a portion of these excess deaths is attributable to natural emissions sources). Thus, our estimates imply that exposure to coal PM_2.5_ was associated with 25% of all PM_2.5_-related Medicare deaths from 2000 to 2008 and with 7% of all PM_2.5_ deaths from 2013 to 2016 (fig. S6).

### Individual EGU contributions to mortality burden

**Fig. 3.**
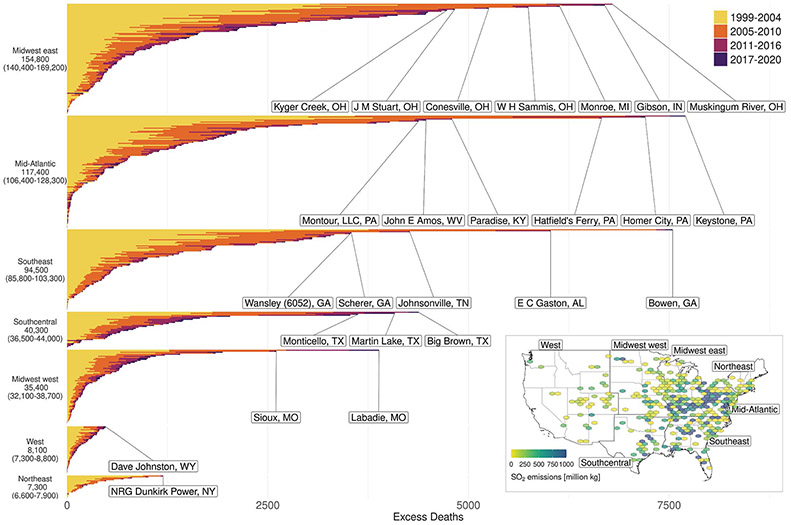
Excess deaths associated with individual coal EGUs from 1999 to 2020. EGUs (*N* = 480) are organized by region to improve interpretability, and the facilities associated with the most deaths are labeled. Inset: total SO_2_ emissions by location from 1999 to 2020 (hexagonal grids may include multiple EGUs) and regional boundaries. Plots were produced in R using ggplot2; spatial information comes from the USAboundaries package.

We identified 138 of the 480 coal EGUs that were associated with >1000 excess deaths across the study period and 10 EGUs that were associated with >5000 deaths (Fig. 3). Although EGUs east of the Mississippi River were associated with the greatest numbers of deaths because of their high emissions and proximity to population centers, each geographical region contained at least one EGU associated with >400 deaths. The distribution of EGU-specific deaths was heavily skewed; 91% of the total deaths were associated with EGUs that accounted for 50% of nationwide coal EGU SO_2_ emissions during the study period. Normalizing excess deaths by energy produced may rank EGUs differently.

**Fig. 4.**
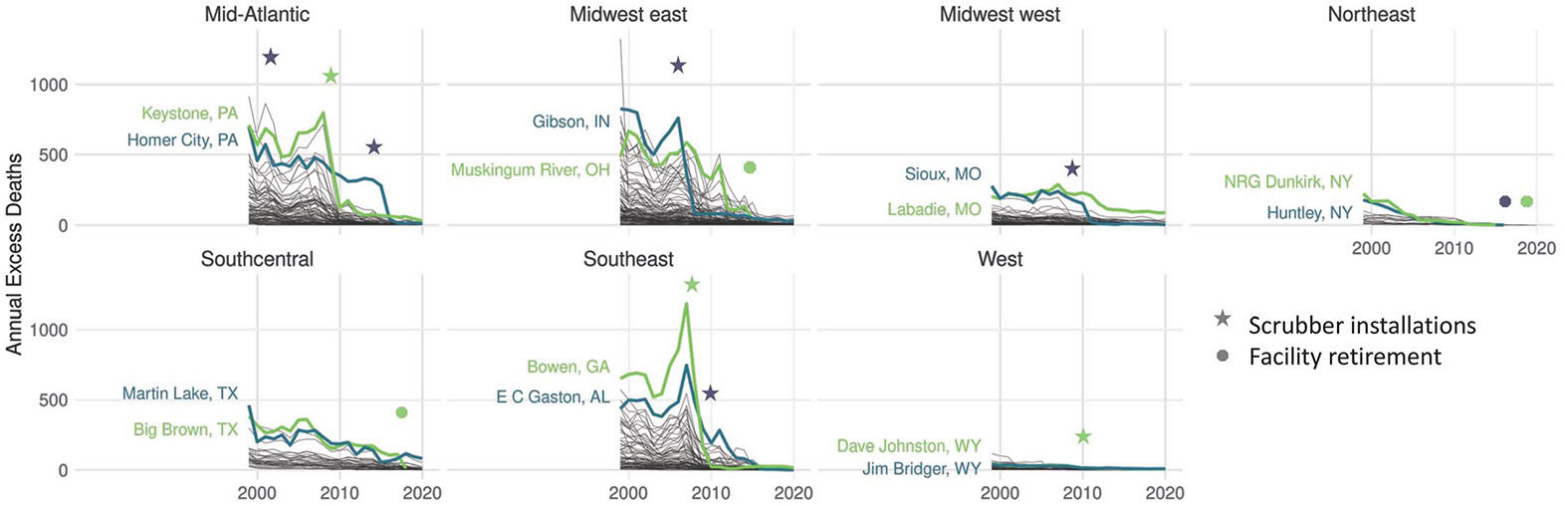
Total annual excess deaths associated with each of the coal EGUs in each region, with the two most harmful facilities highlighted. Scrubber installations designate the earliest year that a scrubber was installed at one or more of each EGU’s units [facility information from (46)]. Plots were produced in R using ggplot2.

Figure 4 shows the temporal trend in the number of deaths associated with each EGU, highlighting the two most harmful coal EGUs within each region. Large declines in the number of deaths corresponded with SO_2_ emission control installations and facility retirements. For example, for the Keystone facility in Pennsylvania, the average annual number of attributable deaths was 640 (95% CI: 580 to 700) before 2008, but declined to 80 (95% CI: 70 to 90) after scrubber installations in 2009 to 2010. We developed an interactive tool to examine individual EGUs and their contributions to state-specific Medicare deaths in relation to SO_2_ emissions control installations and unit retirements (34).

### Sensitivity of results to unmeasured confounding

The stratified Poisson regression for estimating the CRF was chosen based on its use in previous health impact studies of exposure to total PM_2.5_ in the Medicare population. The log-linear CRF implied by the model was chosen to facilitate the source-specific attribution of health impacts, but it may not reflect the true relationship between coal PM_2.5_ and mortality risk across all exposure levels during the study period. Although the stratified Poisson model adjusts for many confounders and has been shown in the context of total PM_2.5_ exposure to be robust to a variety of strategies for confounding adjustment, we cannot rule out the possibility that unmeasured factors related to mortality risk vary systematically with coal PM_2.5_ in a manner not captured by observed characteristics in the model. Using the E-value (34, 35), we found that a potential confounder would need to have an association with both mortality rate and coal PM_2.5_ of 1.125 (lower confidence interval: 1.118) on the RR scale to explain away the association between mortality and coal PM_2.5_.

To explore the potential confounding by air pollution sources other than coal PM_2.5_, we performed several additional sensitivity analyses. We present coal PM_2.5_ RRs from models that adjust for total PM_2.5_, residual PM_2.5_ (total PM_2.5_ minus coal PM_2.5_) as a marker for all other sources, NO_2_ as a marker for primary traffic-related air pollution, and both NO_2_ and residual PM_2.5_ (table S3). Adjusting for total PM_2.5_ attenuated the risk of coal PM_2.5_ substantially, which is consistent with coal PM_2.5_ being captured by the total PM_2.5_ metric. When including residual PM_2.5_ and/or NO_2_, we found a slight attenuation in RR from the main model, and the RR for coal PM_2.5_ remained higher than the RR for total PM_2.5_ found by Wu *et al.* (4). Including markers for other PM_2.5_ sources as confounders introduced important limitations, as explained in the supplementary text. Furthermore, we implemented a “first-differences” analysis of within–ZIP code changes over time to adjust for observed and unobserved differences across ZIP codes (fig. S7). This analysis addresses possible threats to validity caused by confounding differences across different locations, providing strong evidence that areas experiencing larger decreases in coal PM_2.5_ also experienced larger decreases in mortality rates. Results from this analysis support the validity of the primary analysis to quantify the mortality burden with a relative risk adjusted for individual- and ZIP code–level confounders measured throughout the entire study period.

### Sensitivity of results to HyADS characterization of coal PM_2.5_

HyADS rescales air parcel location counts extracted from HYSPLIT to coal PM_2.5_ using a single year’s chemical transport model output, which may introduce errors. Our comparisons (30) of coal PM_2.5_ with coal PM_2.5_ source impacts from year 2006 Hybrid CMAQ-DDM, a full form model (FFM) bias corrected against observations, confirmed that the spatial pattern is well captured and that error and bias are within the typical range of FFMs (although the bias-corrected CMAQ-DDM itself has uncertainty). Because we expected potential errors in coal PM_2.5_ to be smaller in years surrounding the year when the scaling was performed, we retrained the Poisson regression model three times using data only from subsets of the total years available (1999 to 2003, 2004 to 2007, and 2008 to 2016). The estimated RRs from coal PM_2.5_ were comparable but slightly larger than in the main analysis from the 1999 to 2003 and 2004 to 2007 models, with a more pronounced difference in RR from the 2008 to 2016 model (table S3). The change in RR across different periods may be consistent with either genuine changes in risk or deterioration of HyADS’ performance in years further from the year on which the scaling was based (2005).

To explore sensitivity to the process for scaling HyADS to coal PM_2.5_, we estimated the RR and corresponding excess deaths from coal EGUs using unscaled air parcel counts for each ZIP code output by HyADS and found similar estimates of attributable deaths (650,000; 95% CI: 590,000 to 710,000). Comparing coal PM_2.5_ estimates against observed sulfate PM_2.5_ at rural monitors indicated that HyADS may have underestimated exposure and exaggerated exposure declines during the study period (a portion of this decline in coal PM_2.5_ is attributable to decreasing EGU contributions to total US SO_2_ emissions). A sensitivity analysis using a sulfate-adjusted coal PM_2.5_ metric (fig. S8) estimates a mortality RR of 1.0147 (95% CI: 1.0135–1.0158) and 790,000 (95% CI: 720,000–850,000) excess deaths. These findings indicate that, to the extent that HyADS might underestimate coal-derived PM_2.5_ and exaggerate exposure declines, it provides a conservative estimate of the mortality burden associated with exposure to SO_2_ emissions from coal EGUs. Future studies may use newly developed approaches for estimating CRFs that account for uncertainty in air pollution exposure (36).

We used SO_2_ emissions from coal to derive coal PM_2.5_ because of evidence that secondary PM_2.5_ from SO_2_ emissions constitutes most of the ambient PM_2.5_ from coal EGUs during the study period (13, 17, 37). Because SO_2_ emissions and related atmospheric physical-chemical processes that increase ambient PM_2.5_ are correlated with complementary processes of other species, e.g., primary PM_2.5_ and NO_x_, coal PM_2.5_ captures the influence of these other species. Although primary PM_2.5_ emissions are not measured at each EGU, estimated nationwide annual primary PM_2.5_ EGU emissions are correlated (*R*^2^ = 0.97) with measured nationwide annual SO_2_ emissions (38). Sensitivity analyses using observed sulfate and comparisons with alternative modeling strategies revealed broad consistency with the primary analysis, particularly in EGU relative rankings by excess deaths, indicating bounds on uncertainties associated with the diversity of technologies and assumptions available for assessing exposure to EGU SO_2_ emissions.

### Comparison with deaths estimated using a chemistry-transport air quality model

Although it is impossible to directly validate the estimated number of excess deaths attributable to coal PM_2.5_, we compared our results with analogous coal EGU health burdens derived using atmospheric sensitivities from an FFM. Using GEOS-Chem adjoint PM_2.5_ sensitivities (13) and the coal PM_2.5_ RR from the main analysis, we estimated 20,000 (95% CI: 19,000 to 22,000) and 13,000 (95% CI: 12,000 to 14,000) excess deaths in 2006 and 2011, respectively (these years were chosen to span emissions reductions after 2006 and to align with previously published GEOS-Chem adjoint results). These values are comparable, although smaller (especially in 2006) than the excess deaths estimated from coal PM_2.5_ exposure in this study of 35,000 (95% CI: 32,000 to 38,000) and 15,000 (95% CI: 13,000 to 16,000) in 2006 and 2011, respectively. Correlations between the number of deaths assigned to each coal EGU by HyADS and GEOS-Chem adjoint were high (*R*^2^ ≥ 0.85) for all EGUs and for EGUs in most regions (fig. S9 and table S4), and the two models rank ordered EGUs similarly by their associated deaths (fig. S10). Mean differences in nationwide HyADS EGU-specific death estimates relative to the chemical transport model were higher in 2006 (71% for all EGUs) than in 2011 (15%). Although GEOS-Chem adjoint results from the 2 years available are difficult to project to all 22 years of this study, and because chemical transport models, including GEOS-Chem, have uncertainties due to potential bias in emissions inputs, model parameterizations, and meteorology, agreement between the models at levels consistent with previous studies (39) increases confidence in the results reported here.

## Implications

We conducted the longest-term national study to date estimating the excess number of deaths associated with exposure to SO_2_ emissions from US coal EGUs. A key innovation in this study is the combined use of coal EGU-specific exposure estimates and individual-level health data on the same population during the same time period to estimate the mortality burden. This approach has been hampered until now by the limited availability of large-scale health databases and source-specific exposure estimates. Our approach illustrates the utility of deriving air pollution exposure with a combination of dispersion-based and chemical transport models in epidemiological and risk assessment for well-characterized sources.

We found that, over the past two decades in the US, coal PM_2.5_ was associated with 460,000 extra deaths, constituting >22% of total excess deaths attributable to PM_2.5_. We also found that the mortality burden of coal PM_2.5_ has been underestimated using traditional impact assessments that rely on CRFs for total PM_2.5_ mass (13, 16-18, 39-41). The elevated mortality RR associated with annual exposure to coal PM_2.5_ aligns with previous evidence of increased relative health risks associated with coal-related PM_2.5_ or sulfur or sulfate exposure per unit concentration (19-25), although other studies have found little evidence of increased risk related to secondary sulfate PM_2.5_ or PM_2.5_ associated with coal (26, 27). Large decreases in annual deaths across the study period high-light the success of emissions reductions brought about by regulations under the 1990 Clean Air Act Amendments. Although coal use in the US has remained low, global use is expected to increase and plateau by 2025 (8), suggesting the potential for high mortality costs from coal for years to come.

We used SO_2_ emissions from coal to derive coal PM_2.5_; however, we cannot conclude that the portion of ambient PM_2.5_ associated with SO_2_ emissions emitted from coal power plants is more or less harmful than ambient PM_2.5_ from other species emitted from coal power plants. Disentangling the mortality risks of the various PM_2.5_ species emitted from coal EGU emissions is not possible within our modeling framework because of the high correlation between species emitted from coal EGUs such as NO_x_ and primary PM_2.5_. Given how we estimate exposure to “coal PM_2.5_,” our finding of a higher mortality risk of exposure to coal PM_2.5_ relative to other PM_2.5_ suggests the potential for population health benefits of reducing SO_2_ emissions from coal power plants, for example, by installing emissions control devices or shutting coal facilities completely. Full separation of the health impacts of various emitted species from coal EGUs is of additional interest to policy-makers because of the varying technologies available to reduce EGU emissions of specific pollutants, and it should be considered in future studies.

HyADS benefits from well-characterized source locations and emissions, along with the relatively slow atmospheric transformation of emitted SO_2_ to particulate sulfate. Expanded incorporation of information from observation and chemical transport model–based source apportionment techniques in reduced complexity models may enable linkages between emitted species beyond SO_2_, atmospheric processes, exposure, and health outcomes. Although source-specific PM_2.5_ cannot be directly measured, observation-based receptor methods for PM_2.5_ source apportionment (42) could provide an approximate ground truth (albeit with their own uncertainties) for evaluating modeled source-specific exposure. Advanced sensitivity approaches incorporated within chemical transport models, such as GEOS-Chem Adjoint used here, and sensitivity methods such as the direct decoupled method (DDM) (43) or the integrated source apportionment method (ISAM) (44) offer model-based approaches that more explicitly incorporate atmospheric chemistry and physics. Expanding computational capacity will make comparisons with these types of models in applications with many sources increasingly feasible.

These results advance the growing body of evidence showing varying toxicity of PM_2.5_ originating from different sources. Although the US and other countries continue to regulate total ambient PM_2.5_ concentrations, entities such as the EPA Clean Air Scientific Advisory Committee have specifically cited a need for research to assess health effects associated with changes in PM_2.5_ composition and sources over time as an important consideration for future PM_2.5_ policy assessments (45). Our findings have implications for current air pollution risk assessments, which incorrectly assume equal toxicity for ambient PM_2.5_ from all sources and for all locations. The research platform that we used to quantify exposure associated with individual coal EGUs, which accounts for pollution transport and location relative to population centers, can support more efficient regulatory efforts by producing targeted evidence of how individual EGU sources contribute to the existing health burden.

## Supplementary Material

Supplementary Methods

Reproducibility Checklist
