# Supplementary material for: Mortality risk from United States coal electricity generation
Source: Science. Author manuscript; Available in PMC 2024 Feb 16. (PMC10870829; doi:10.1126/science.adf4915)
Supplement: Supplementary Methods [file NIHMS1963859-supplement-Supplementary_Methods.pdf]

Science
AAAS

Supplementary Materials for

Mortality risk from United States coal electricity generation

Lucas Henneman *et al*.

Corresponding author: Lucas Henneman, lhennem@gmu.edu

Science **382**, 941 (2023)
DOI: 10.1126/science.adf4915

The PDF file includes:

Materials and Methods
Supplementary Text
Figs. S1 to S10
Tables S1 and S2
References

Other Supplementary Material for this manuscript includes the following:

MDAR Reproducibility Checklist

Materials and Methods

Our methods comprise three main tasks. We 1) estimated annual exposure to $PM_{2.5}$ from coal electricity generating unit (EGU) SO_2 emissions ("coal $PM_{2.5}$") in each ZIP code; 2) estimated the concentration response function (CRF) describing the association between coal $PM_{2.5}$ and all-cause mortality using an established epidemiological model that adjusts for multiple measured confounders; and 3) estimated the number of deaths in the Medicare population associated with SO_2 emissions from each individual coal EGU facility and in total across all facilities using the estimated CRF.

Estimating coal $PM_{2.5}$ exposure from coal EGU SO_2 emissions

EPA's Air Markets Program Data (54) contains detailed information on 1,237 coal EGUs at 480 facilities in operation for at least one month between 1999 and 2020 (each facility may contain multiple EGUs). For each coal EGU, we applied the HYSPLIT with Average Dispersion (HyADS) model to estimate annual exposure to $PM_{2.5}$ directly attributable to coal EGU SO_2 emissions. In previous work, we demonstrated the importance of accounting for inter-annual variability in meteorology when simulating coal source impacts across years (30). Employing the HyADS model for individual EGU impacts ensures our coal $PM_{2.5}$ tracks annual exposure variability absent from modeling approaches that assume constant meteorological conditions. We have also shown that HyADS performs well against more complex models and is appropriate for identifying population impacts of emissions from coal EGUs. While detailed descriptions and evaluations of HyADS exist elsewhere (31, 32, 52) including in previous epidemiological studies (23, 29, 55), we provide an overview here.

Starting at each EGU's latitude, longitude, and stack height, we applied the HYSPLIT (56) transport and dispersion model to track air parcels traversing the atmosphere. We initiated 100 parcels at each unit four times per day and tracked the parcels for seven days. After removing the parcel locations in the first hour after emissions to account for unreasonably high near-source concentrations, we summed all parcel locations from a given unit over a 36-km grid by month and multiplied the summed field by each EGU's monthly SO_2 emissions. Uncertainty in SO_2 emissions reported by EPA is low, with a relative accuracy of 3% on average (57). These unitless source impacts fields are highly correlated with $PM_{2.5}$ source impacts fields from all U.S.A. coal emissions as calculated using a Hybrid Chemical Transport-Receptor Modeling Approach (58) in 2005 and 2006. Uncertainty in HyADS single EGU source impacts evaluated against GEOS-Chem (a chemical transport model) adjoint $PM_{2.5}$ sensitivities to annual SO_2 emissions is within typical performance of air quality models (41). We employed year 2005 Hybrid CMAQ-DDM (58) (derived output from a second chemical transport model) source impacts fields to post-process raw annual HyADS output into coal $PM_{2.5}$, as described previously (30, 52). Using HyADS, we estimated annual coal $PM_{2.5}$ exposure for each ZIP code for each of 1,237 coal EGUs in operation across 1999–2020 and aggregated the contributed exposure to 480 coal facilities.

Absent any direct observations of 1) total coal $PM_{2.5}$ pollution exposure or 2) $PM_{2.5}$ source impacts from individual power plants, our evaluations of HyADS here and in the citations above have taken two forms. HyADS' favorable comparison with Hybrid CMAQ-DDM $PM_{2.5}$ sensitivities to coal-related emissions in 2006 supports the ability of the emissions-weighted trajectory-based model to approximate coal $PM_{2.5}$ exposure fields in a single year (31). Using GEOS-Chem adjoint simulations as the closest available way to estimate the "ground truth" of individual facility coal $PM_{2.5}$ source impacts, we find that a) emissions and location play a

dominating role in the characterization of coal source impacts; b) HyADS can approximate source impacts; and c) the extent of approximation of GEOS-Chem adjoint sensitivities varies across 2006 and 2011 (30). In Henneman et al. (2019) (32), we identified the importance of incorporating concurrent wind fields when modeling source-specific exposure fields or impacts using HyADS. In comparisons with both SO_x and SO_x+NO_x GEOS-Chem adjoint sensitivities in 2006 and 2011 in Henneman et al. (2023) (52), we established support for a) HyADS' ability to characterize coal $PM_{2.5}$ source impacts; b) variability across years with different emissions mixes; and c) the importance of geographically disaggregated evaluation of coal $PM_{2.5}$ source impacts.

Coal $PM_{2.5}$ as presented here represents total $PM_{2.5}$ associated with coal EGU SO_2 emissions—we did not differentiate the portion of coal $PM_{2.5}$ that is sulfate or other chemicals that may be either emitted simultaneously or associated with emitted SO_2 in the atmosphere, such as metals or organic gases (59). Insofar as other coal source impacts—e.g., ozone from NO_x emissions or trace emissions of metals—are spatially and temporally correlated with coal $PM_{2.5}$, a portion of the reported deaths may be attributable to these other atmospheric byproducts of coal EGUs. Previous risk assessments have identified emitted SO_2 as the largest pollutant contributor to health risk from coal EGUs because of its contribution to sulfate $PM_{2.5}$ (13, 17, 43), although ambient exposure to nitrate $PM_{2.5}$, NO_2, and ozone from NO_x emissions have additional health impacts. HyADS-estimated coal $PM_{2.5}$ showed better agreement with population-weighted $PM_{2.5}$ attributable to EGU SO_2 emissions than $PM_{2.5}$ attributable to both coal EGU SO_2 and NO_x emissions (52).

Medicare and demographic data

We employed annual ZIP code-level all-cause mortality data from U.S.A. Medicare beneficiaries from 2000 to 2016, available through a data use agreement with the Center for Medicare and Medicaid Services. We augmented these data with population demographic data from the U.S.A Census Bureau and the Center for Disease Control and Prevention Behavioral Risk Factor Surveillance System (BRFSS), as well as meteorological data from Google Earth Engine's Gridmet. All data was mapped to postal ZIP codes. The Medicare and confounders dataset was used previously by Wu et al. (2020) (4).

Estimating concentration response function

We estimated the association between total number of annual deaths in the Medicare population and annual average coal $PM_{2.5}$ using a longitudinal study design. We applied a stratified Poisson regression model previously deployed in the Medicare population in a study of long-term associations with total mass $PM_{2.5}$ (4). The model considers annual coal $PM_{2.5}$ as a time-varying exposure and models the all-cause mortality rate in a ZIP code at a given follow-up year and calendar year, adjusted for many confounding factors. Specifically, following the approach of Wu et al. (2020) (4), we stratified the model by follow-up year and four individual level characteristics (age in 5-year categories, race/ethnicity [White, Black, Asian, Hispanic, Native American, and other], gender, and Medicaid eligibility) and controlled for 14 ZIP code-level covariates, a dummy region variable, and a dummy calendar year variable. ZIP code-level covariates included mean BMI and smoking rate from the BRFSS (%); percent Hispanic, percent Black, median household income, median home value, percent of residents living in poverty, percent of residents graduating high school, population density, and percent of residents who own a home from the U.S.A Census; and summer (June-September) and winter (December-February) average maximum daily temperature and relative humidity from Google Earth Engine's Gridmet, which is averaged to ZIP codes from 4-km grids (variables described in **table**

S1). An indicator variable for each region (Northeast, South, Midwest, and West) controlled for residual spatial confounding. An indicator variable for each year controlled for residual temporal confounding, including, for example, regulations that reduced emissions of other air pollutants from power plants or changes in underlying mortality rate.

The log-linear stratified Poisson model captured the average relationship between mortality and coal $PM_{2.5}$. Consistent with its previous application, the possibility of heterogeneity with respect to time, space, or population features was not reflected in the model. The analysis in Wu et al. (2020) found consistent results from the stratified Poisson model and three additional models using more sophisticated causal-inference techniques, which we did not pursue here. Further details of the model specification are described in Wu et al. (2020) (4).

To estimate 95% confidence intervals while accounting for the possibility of residual spatial autocorrelation not captured by the inclusion of the confounders specified above, we applied the m-by-n blocked bootstrap method used by Wu et al. (2020) (4). To do so, we sampled 374 ZIP codes from the entire dataset 500 times with replacement and recalculated the model; this number of ZIP codes was chosen based on a common rule of thumb supported by theoretical results of the m-by-n blocked bootstrap (60). Large sample 95% confidence intervals were calculated using the bootstrapped standard error estimates for each model parameter.

Coal EGU mortality burden assessment

We calculated total numbers of deaths associated with each coal EGU in each year using equation (1). This formulation follows from a typical risk assessment design with an incremental reduction in exposure $\Delta E_{t,u}^{z}$ in time *t* from EGU u in ZIP code *z*:

$$deaths_{t,u} = \sum_{z=1}^{Z} {M_0}_t^z \times \left(e^{\beta_E \times \Delta E_{t,u}^{z}} - 1\right) \times P_t^z \quad (1)$$

Where β_E is the relevant regression parameter from the stratified Poisson model, ${M_0}_t^z$ is the baseline mortality rate (deaths divided by Medicare enrollees) observed in each year and ZIP code, and P_t^z is the number of Medicare enrollees in a given ZIP code and year. This formulation is analogous to the linear approach described by Nasari et al. (2016) (61) and has been employed in similar forms in many risk assessment tools such as EPA's BenMAP program and the Global Burden of Disease study (61-63). The approach calculates the excess number of deaths in a given year relative to a setting where there were no emissions from EGU u during year t. Estimates of $deaths_{t,u}$ are calculated using the mean and 95% confidence interval of the Poisson model hazard ratio. For 2018–2020, when no Medicare data are available, we used equation (1) with each ZIP code's average mortality rate ${M_0}_t^z$ and enrollees P_t^z during the years 2014–2016.

Risk assessments in air pollution often assume a baseline exposure below which no health outcomes occur (61). The baseline level for coal $PM_{2.5}$ is 0 (i.e., there are no natural sources). We calculated incremental deaths associated with emissions from individual EGUs (i.e., for $\Delta E_{t,u}^{z}$, the portion of the coal $PM_{2.5}$ exposure attributable to a single EGU's emissions in year t to ZIP code z). A complementary approach, wherein all coal $PM_{2.5}$ exposure is removed simultaneously (i.e., substituting $\sum_{u=1}^{U} \Delta E_{t,u}^{z}$ for $\Delta E_{t,u}^{z}$) would produce higher total deaths attributable to coal $PM_{2.5}$ (results differ by ~2%). Because the differences are small and incremental removal of exposure from one unit at a time is more relevant to actual conditions, we presented deaths as the sum of deaths associated with individual EGU's annual coal $PM_{2.5}$, $\Delta E_{t,u}^{z}$. We presented absolute deaths calculated for each facility (each facility may contain multiple EGUs), focusing on relative comparisons across facilities, regions, and years, given that the specific number of deaths associated with any source in an air pollution risk assessment is impossible to independently verify.

Total $PM_{2.5}$ mortality burden assessment

Since coal $PM_{2.5}$ represents a fraction of total $PM_{2.5}$ mass, we calculated the proportion of excess deaths associated with total mass $PM_{2.5}$ that can be attributed specifically to coal $PM_{2.5}$. To do this, we applied the risk assessment design above using the total $PM_{2.5}$ in place of $\Delta E_{t,u}^{z}$ and the RR found by Wu et al. (2020) (4) for the Medicare population. This approach assumes a log-linear relationship across all levels of $PM_{2.5}$ concentrations and zero threshold. While the approach is consistent with previous studies, it does not differentiate anthropogenic from natural sources.

Online data exploration tool

The Coal Pollution Impacts Exploration (CPIE) platform is based on pilot work conducted as part of the U.S.A. Environmental Protection Agency's EmPOWER Air Data Challenge. A preliminary prototype was created using the prototyping platform Figma (figma.com) and tested with users representing a range of experience in air quality to obtain feedback on function, layout, and usability. Feedback from these early iterations informed development of the CPIE platform using the JavaScript library D3.js.

Computing notes

HyADS was run with the disperseR R package (https://github.com/lhenneman/disperseR). We ran the Poisson model with the *gnm* R package.

Supplementary Text

Impacts not accounted for in this study

The public health impacts tallied in this work represent a portion of the total impacts from coal EGUs in the U.S.A. The premature mortality counts were limited to Medicare beneficiaries, which account for most Americans 65 years of age and older. Our analysis did not include other age groups or residents of other countries impacted by U.S.A. coal emissions such as Canada and Mexico. In addition, coal EGUs contribute positive and negative externalities (e.g., societal benefits such as provision of energy and local job creation; public health risk, ecological harm, and environmental injustice from mining, water discharge, and coal transport; and climate impacts) that were not considered in this study. Health impacts of EGU NO_x and primary $PM_{2.5}$ are not included in the calculations except where exposure and health impacts from these species are highly correlated in time and space with SO_2-derived coal $PM_{2.5}$. While we sought to quantify and limit uncertainties associated with the findings here by using high-quality study elements, the death values are impossible to independently verify.

Stability of Medicare mortality rates

To assess the extent that variability in Medicare mortality rates may have influenced the study findings, we present here the annual mortality rates for 482 large ZIP codes (over 1000 Medicare enrollees) with the top 5% change in coal $PM_{2.5}$. Death rates in most of these ZIP codes decreased over time and show year-to-year variability, but they are generally stable at around 0.05 deaths/enrollee (**fig. S5**).

Sensitivity to unmeasured confounding

To assess the potential for the unmeasured confounding to bias the model results, we calculated the E-value for the main Poisson model (35, 36). The E-value for the main analysis is 1.125 (lower confidence interval: 1.118), meaning that in order for an unmeasured confounder to nullify the association between mortality and coal $PM_{2.5}$, that unmeasured confounder would need to yield a 12.5% increase in mortality after having adjusted for all the other measured

confounders. The large E-value relative to the coal $PM_{2.5}$ RR provides evidence that unmeasured confounding not attributable to any other adjustment factors in the model would have to be very strong to account for the significant relative risk.

Sensitivity analyses that include additional confounding adjustment for other pollution sources

We re-trained the Poisson model using additional ambient air pollution exposure as potential confounders of the association between coal $PM_{2.5}$ and mortality (**table S3**). The new confounders included total $PM_{2.5}$ concentrations, "residual $PM_{2.5}$" calculated as the difference between total $PM_{2.5}$ and coal $PM_{2.5}$, and annual NO_2 concentrations (64). These were chosen as markers for air pollution from sources other than coal EGUs, but they are associated with their own limitations. Residual $PM_{2.5}$, for example, is limited because the uncertainty structure is difficult to quantify (the estimates of the two component terms were developed with different models, and some of the values are negative). Since coal $PM_{2.5}$ is part of total mass $PM_{2.5}$, adjustment for total mass might absorb some of the estimated impact of coal $PM_{2.5}$ into the confounding adjustment. NO_2 presents a similar issue—while NO_2 concentrations are often used as a marker for traffic sources, EGUs are a traditionally large NO_2 source, suggesting a similar potential for misattributing the effect of interest to another variable that is a consequence of the exposure of interest.

Sensitivity analyses to assess potential influence of varying bias in coal $PM_{2.5}$ over time

The HyADS model accounts for air transport and dispersion but is limited by an implicit assumption that the statistical relationship between EGU SO_2 emissions and coal $PM_{2.5}$ is constant for given meteorological conditions, a known simplification during this period (13, 53). Since most coal $PM_{2.5}$ is expected to be sulfate $PM_{2.5}$, and most U.S.A. sulfate over this period is attributable to coal SO_2 emissions (40), we expected agreement between coal $PM_{2.5}$ and observed ambient sulfate $PM_{2.5}$. We found that the coal $PM_{2.5}$ trend in each region is similar to the observed sulfate $PM_{2.5}$ trend at rural IMPROVE network monitor locations. The IMPROVE network was designed to assess visibility impairments by anthropogenic emissions in Class 1 federal areas (65). By applying observations from this network as a surrogate for coal $PM_{2.5}$ in the sensitivity analysis, we assumed that the influence from urban, non-coal EGU SO_2 sources is small on this primarily rural network. We found higher agreement in both magnitude and correlation in the early portion of the study period (**fig. S8**), when coal EGUs explained a greater fraction of U.S.A. sulfur emissions (EGUs contributed between 65% and 70% of annual U.S.A. SO_2 emissions in 1999–2015 and under 55% in 2017–2020 (40)). Thus, taking the IMPROVE monitoring sites as a proxy for trends in regional coal $PM_{2.5}$, our estimates of coal $PM_{2.5}$ exposure may underestimate true exposures and indicate a faster decline in exposure relative to actual changes in exposure to $PM_{2.5}$ related to coal EGU SO_2 emissions during the study period.

We explored the influence of the difference between HyADS-estimated coal $PM_{2.5}$ and observed sulfate $PM_{2.5}$ in the statistical scaling used by HyADS by scaling the HyADS coal $PM_{2.5}$ by the observed mean sulfate $PM_{2.5}$ in each region at IMPROVE monitoring sites (**fig. S8**) and retraining the stratified Poisson model. This rescaling implies a slower decline in coal $PM_{2.5}$ than used in the main analysis. Under the rationale that it is unlikely that coal $PM_{2.5}$ decreased more slowly than observed sulfate at IMPROVE monitors (as EGU emissions are still estimated to account for over half of U.S.A. sulfur emissions in 2017–2020), this assumed slower decline represents a conservative characterization of the decline in coal $PM_{2.5}$. Sulfate-adjusted coal $PM_{2.5}$ had a higher mean (1.45 $\mu g\ m^{-3}$) and maximum ZIP code value (12.72 $\mu g\ m^{-3}$) than unadjusted coal $PM_{2.5}$. The resulting sulfate-adjusted coal $PM_{2.5}$ RR was 1.0147 (95% CI: 1.0135–1.0158) per 1 $\mu g\ m^{-3}$, which is slightly larger than the base model RR. Using the sulfate-

adjusted RR and sulfate-adjusted total $PM_{2.5}$ concentrations, we estimated 790,000 (95% CI: 720,000–850,000) excess deaths. These early death estimates are larger than those in the primary analysis because of both the higher RR and the slower reduction in sulfate-adjusted coal $PM_{2.5}$ across the study period than unadjusted coal $PM_{2.5}$.

To further explore the influence of the scaling in HyADS to coal $PM_{2.5}$, we estimated RR and corresponding excess deaths from coal EGUs using unscaled air parcel counts in each ZIP code. The RR from this analysis is not interpretable relative to pollutant concentrations, but the number of excess deaths is calculated in a comparable way to the main analysis using the same un-scaled air parcel counts. This analysis found a similar, yet slightly larger number of excess deaths across the study period (660,000; 95% CI: 600,000–730,000), establishing that uncertainty associated with the scaling to a single year's chemical transport model output does not dominate the HyADS modeling process.

We tested the influence of training the Poisson model across different years of the study to understand how bias in coal $PM_{2.5}$ that may increase in years further from the year it was calibrated with the full complexity chemical transport model (year 2005) influenced the estimated RR (**table S3**). A model trained only on data from 2005–2007 yielded a RR similar to but slightly larger than the main model.

<u>Medicare mortality rate changes in areas with relatively large changes in coal $PM_{2.5}$ over time</u>

As an alternative to the stratified Poisson model, we also employed a first-differences approach similar to Henneman et al. (2019) (23) to assess the extent to which within-ZIP code changes in mortality rates over time were associated with concurrent ZIP code decreases in coal $PM_{2.5}$. Key features of this analysis were derived from comparing locations with themselves over time and include 1) ability to adjust for unobserved ZIP code confounders that do not change over time; and 2) reduced reliance on the HyADS post-processing to convert air parcel locations to coal $PM_{2.5}$, since only the rank order of the temporal change in exposure is used. First, for a given year (t), each ZIP code is classified into one of M=20 quantiles according to its change in HyADS from year 2000 to year *t*, denoted with Q_{qt}. Similarly, the change in mortality rate from year 2000 to year *t* is calculated for each ZIP code, denoted R^{Δ}_{t-2000}. We used equation (2) to estimate the association between the change in mortality rate and the change in coal $PM_{2.5}$ to assess whether locations with more improved exposure exhibited the starkest mortality changes. We controlled for all the baseline covariates in the primary analysis in 2000 ($X_{c,2000}$) and their change since 2000 ($X^{\Delta}_{c,t-2000}$; all covariates in equation (2) are scaled by subtracting their means).

$$R^{\Delta}_{t-2000} = \sum_{q=1}^{M} \beta_q Q_{qt} + \sum_{c=1}^{C} \beta_c X_{c,2000} + \sum_{c=1}^{C} \beta^{\Delta}_c X^{\Delta}_{c,t-2000} + \varepsilon \qquad (2)$$

In **fig. S7**, we plotted β_q against Q_{qt} beginning in 2008, the year that coal $PM_{2.5}$ began decreasing systematically across the U.S.A., and repeated the model for subsequent years. Quantiles denoted by larger numbers denote areas with larger changes in exposure since 2000, so negative slopes suggest that areas that saw the largest decrease in coal $PM_{2.5}$ exposure also saw the largest decreases in mortality rates, controlling for all covariates in equation (2). Linear trend lines have been added to **fig. S7** to aid interpretation. Similar to our previous work, we do not present the slope of these lines, but instead focus on the negative slope to affirm that death rates decreased faster on average with larger coal $PM_{2.5}$ exposure decreases.

As slopes of β_q vs. Q_{qt} were negative in all years, with statistically significant ($p < 0.05$) negative trends in 6 out of 8 years presented, the analysis suggests that areas that experienced larger decreases in coal $PM_{2.5}$ also experienced larger decreases in mortality rates (**fig. S7)**.

Role of baseline mortality rate and coal $PM_{2.5}$ in excess mortality estimates

Three elements dictate annual excess deaths from coal $PM_{2.5}$: the RR calculated by the Poisson model, coal $PM_{2.5}$ exposure, and baseline mortality rates. In **Fig. 2**, we assessed the influence of the RR by comparing the number of excess deaths from coal $PM_{2.5}$ calculated assuming multiple RRs from this study and the literature. In **fig. S4**, we show the relative influence of changing coal $PM_{2.5}$ exposure and changing baseline mortality rates on changes in excess mortality since 1999 under two hypothetical scenarios. In the first, we isolated the influence of changing coal $PM_{2.5}$ exposure by holding baseline mortality constant at 1999 levels. In the second, we isolated the influence of baseline mortality rates by holding coal $PM_{2.5}$ constant at 1999 levels. Most of the change in annual excess deaths from coal $PM_{2.5}$ comes from the reduction in coal $PM_{2.5}$ exposure (dashed line in **fig. S4**). Because of changing mortality rates, annual excess deaths from coal $PM_{2.5}$ would have increased slightly across the period if coal $PM_{2.5}$ remained at 1999 levels (solid line in **fig. S4**).

GEOS-Chem adjoint comparison

GEOS-Chem adjoint sensitivities are provided as aggregate annual average population-weighted $PM_{2.5}$ attributable to SO_2 emissions perturbations anywhere in the U.S.A., and computed for the years of 2006 and 2011 based on EPA's National Emissions Inventory of 2005 and 2011, respectively (13). To estimate deaths contributed by each coal EGU, we applied equation (1) for each EGU in 2006 and 2011 using the concentration unit sensitivities in place of $\Delta E_{t,u}^{z}$ and the national Medicare death rate and population in place of ${M_0}_t^z$ and P_t^z, respectively. We used the RR from the stratified Poisson model developed in this work in the GEOS-Chem adjoint risk assessment. Due to the way in which GEOS-Chem adjoint sensitivities are calculated, this comparison assumes that mortality patterns in the general population distribution are correlated with those in the Medicare population. Comparisons of EGU facilities by the number of associated deaths modeled by GEOS-Chem and HyADS were grouped by region, with linear relationships between them summarized using linear regressions (**fig. S9** and **table S4**). Most of the region-specific comparisons fell within previously published comparison statistics of widely used reduced complexity air quality models that calculate damages from individual sources (41); the differences are likely attributable to year- and region-specific differences in transport and chemistry parameterizations in the HyADS and GEOS-Chem models and differences in spatial grid resolution.

Fig. S10 compares how both GEOS-Chem and HyADS ranked facility impacts relative to what would be learned by considering only SO_2 emissions magnitudes. Deviations from the 1:1 line in this comparison showed consistent direction for facility rankings by HyADS coal $PM_{2.5}$ and GEOS-Chem adjoint sensitivities; for example, in the Mid-Atlantic and Midwest East regions, both modeling efforts ranked facilities by associated mortality impacts higher than their ranking by SO_2 emissions. Both models ranked facilities relatively lower than the emissions-only ranking in the Southcentral and West regions. This comparison highlights that HyADS and GEOS-Chem adjoint sensitivities identify similar regions in which facility rankings by SO_2 emissions differ from their rankings by associated deaths, and the two models identify similar relationships between SO_2 emissions ranking and associated death ranking.

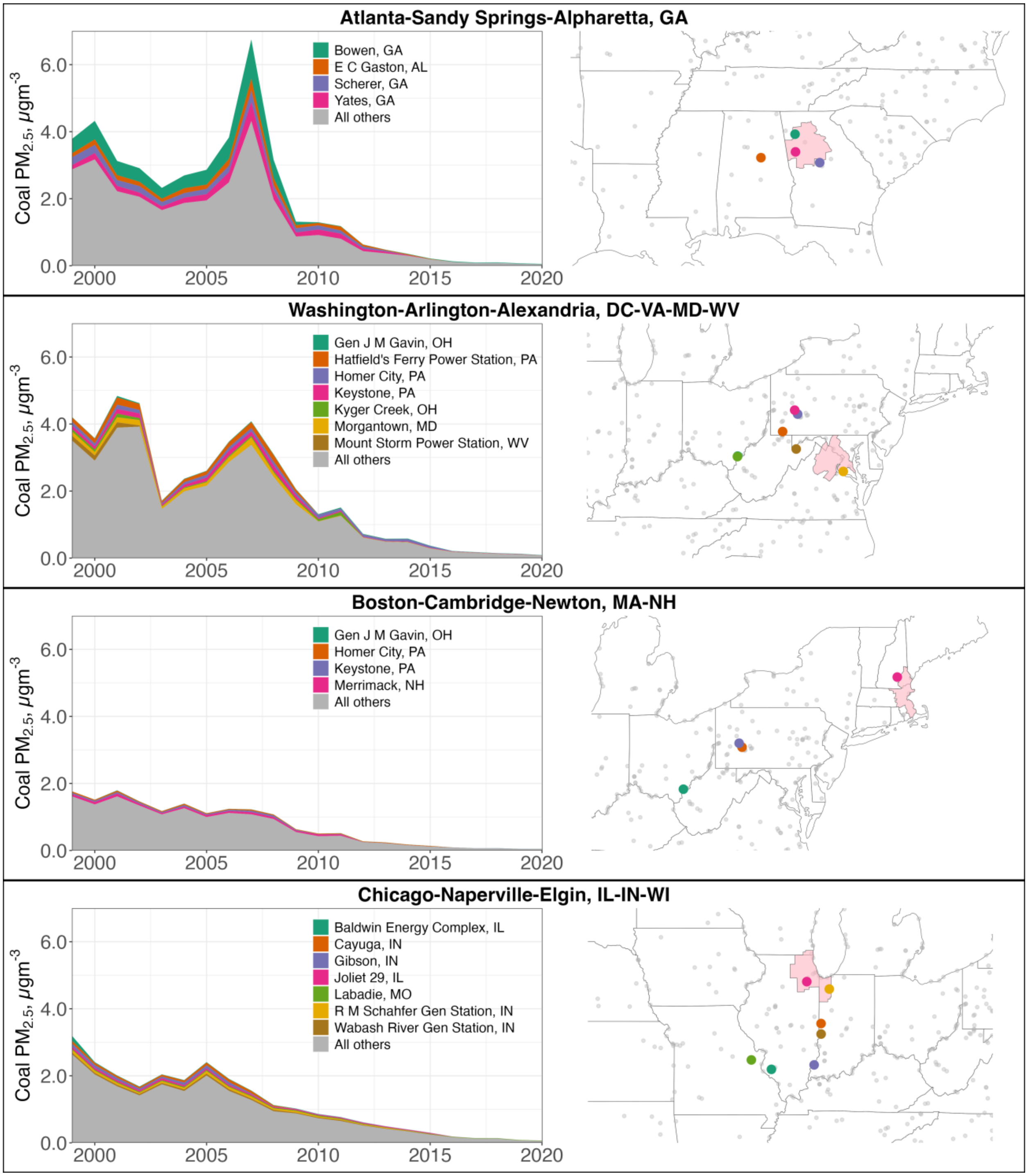

Fig. S1. EGU facility coal $PM_{2.5}$ exposure in four metropolitan statistical areas. Named facilities contributed more coal $PM_{2.5}$ to each metropolitan area than all other facilities in at least one year across the study period. Other coal facilities are shown in grey.

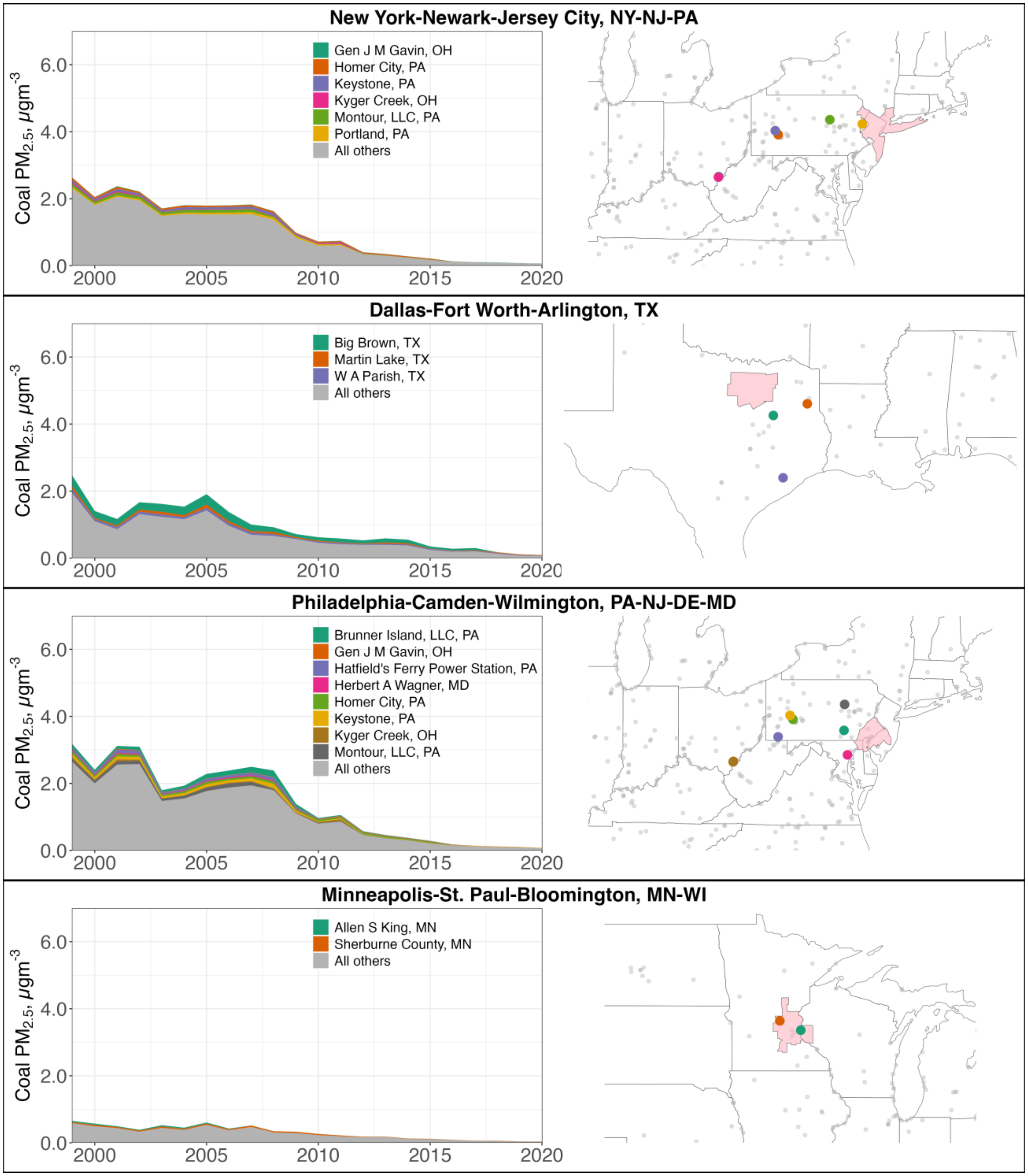

Fig. S2. EGU facility coal $PM_{2.5}$ exposure in four metropolitan statistical areas. Named facilities contributed more coal $PM_{2.5}$ to each metropolitan area than all other facilities in at least one year across the study period. Other coal facilities are shown in gray.

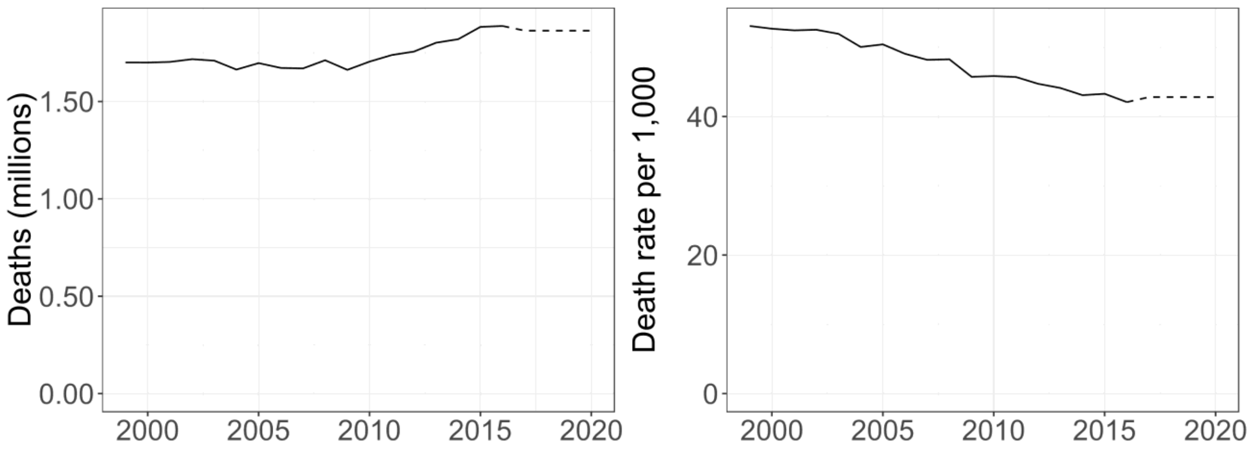

Fig. S3. Annual deaths and death rates in the Medicare population. Dashed lines denote extrapolated data (2017–2020).

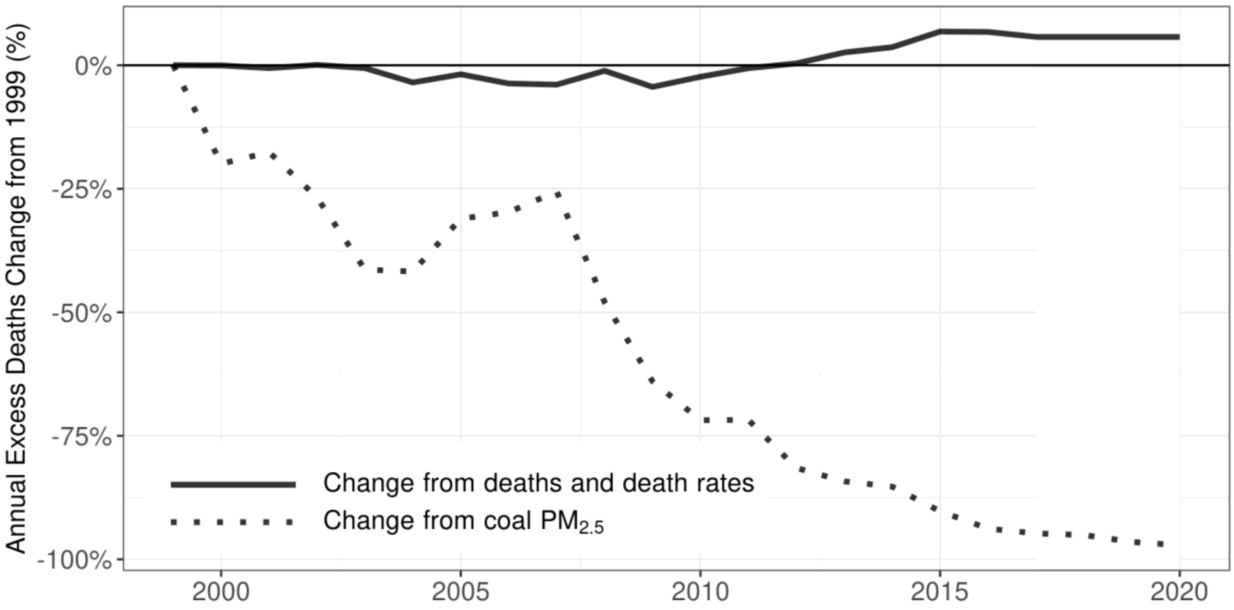

Fig. S4. Percent change from 1999 in annual excess deaths from coal $PM_{2.5}$ attributable to changes in the number of deaths and death rates in each ZIP code (solid line) and changes in coal $PM_{2.5}$ (dotted line).

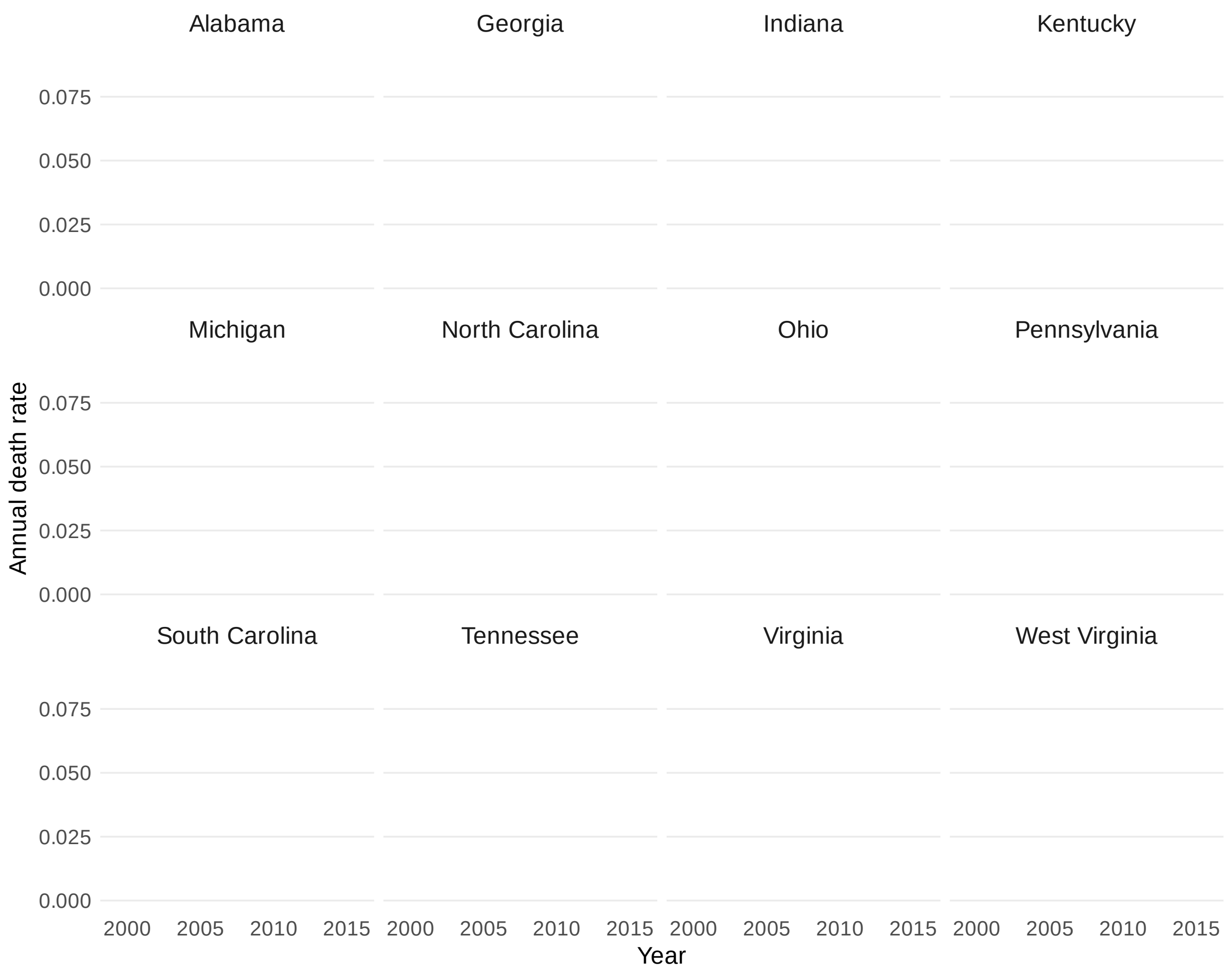

Fig. S5. Changes in mortality rate in 482 large ZIP codes (over 1000 Medicare enrollees) that saw the top 5% change in coal $PM_{2.5}$.

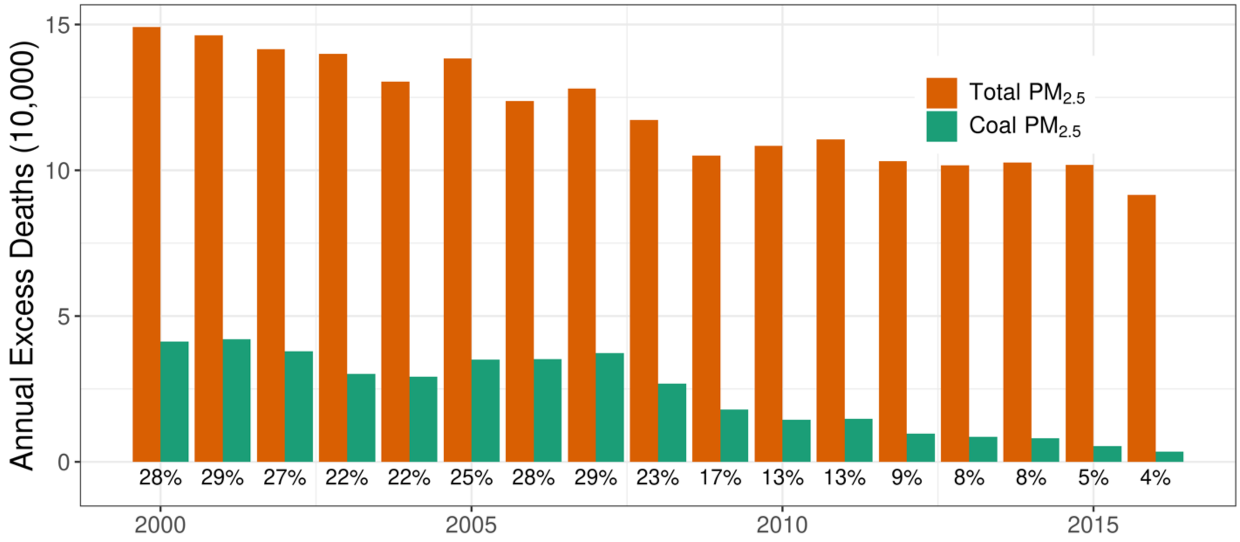

Fig. S6. Deaths associated with exposure to total $PM_{2.5}$ and coal $PM_{2.5}$ by year. Percentages reported at the base of the plot represent the percent of total $PM_{2.5}$ deaths from coal $PM_{2.5}$. Total $PM_{2.5}$ deaths are calculated using the $PM_{2.5}$ RR reported by Wu et al. (2020) (4) for the Medicare population and the $PM_{2.5}$ concentration fields used in that paper, available from 2000 to 2016.

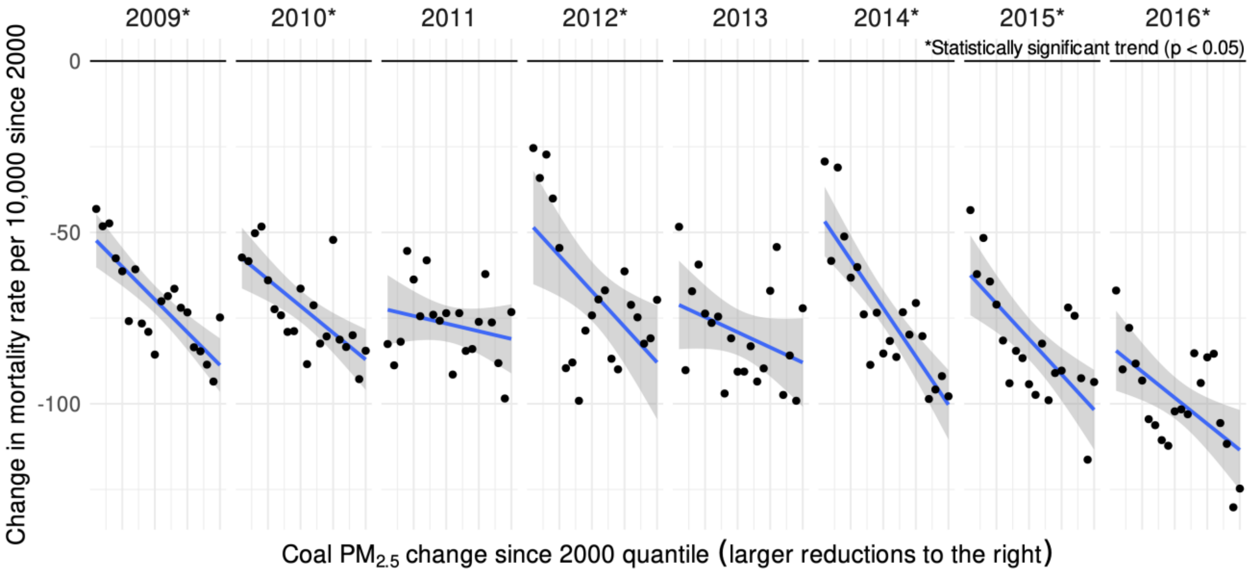

Fig. S7. Changes in mortality rate per 10,000 person-years (beneficiaries for all Mortality) in ZIP codes that fall in each exposure change quantile ($-\beta_q$) estimated with Model 2. Negative values on the vertical axis denote decreases in health outcome rates. Panel labels (2009, 2010, …, 2016) denote years of comparison to 2000 (e.g., differences in mortality rate in 2009 relative to 2000, differences in mortality rate in 2010 relative to 2000, etc.).

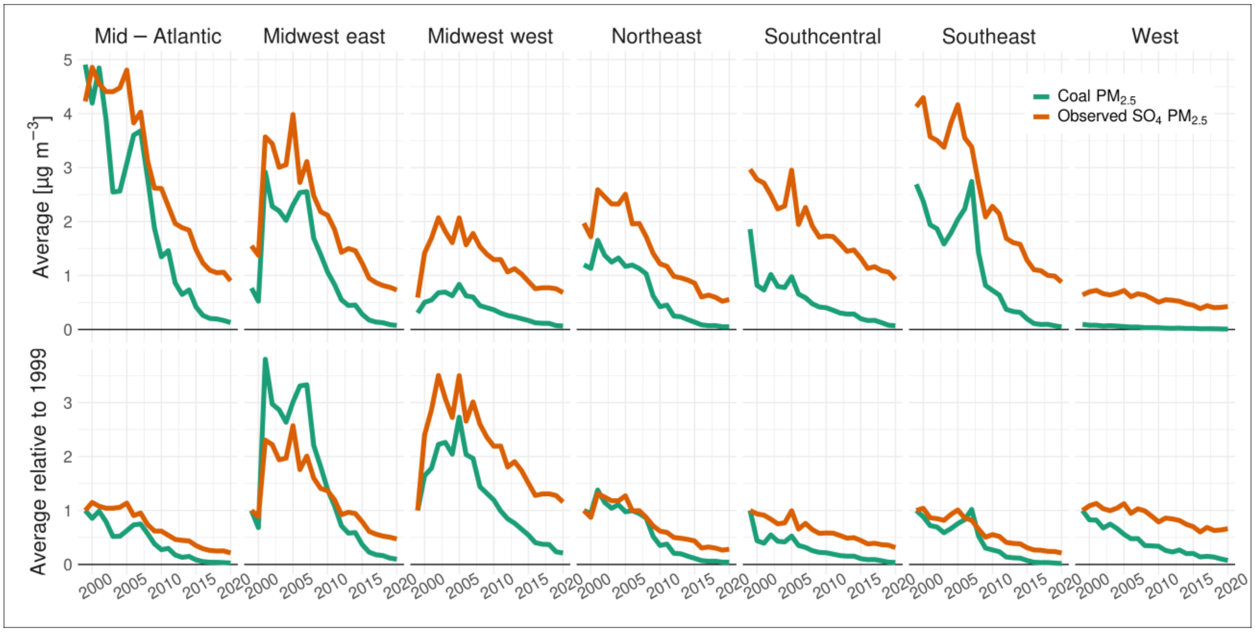

Fig. S8. Coal $PM_{2.5}$ and observed sulfate at IMPROVE monitors averaged by region (top) and relative to 1999 (bottom).

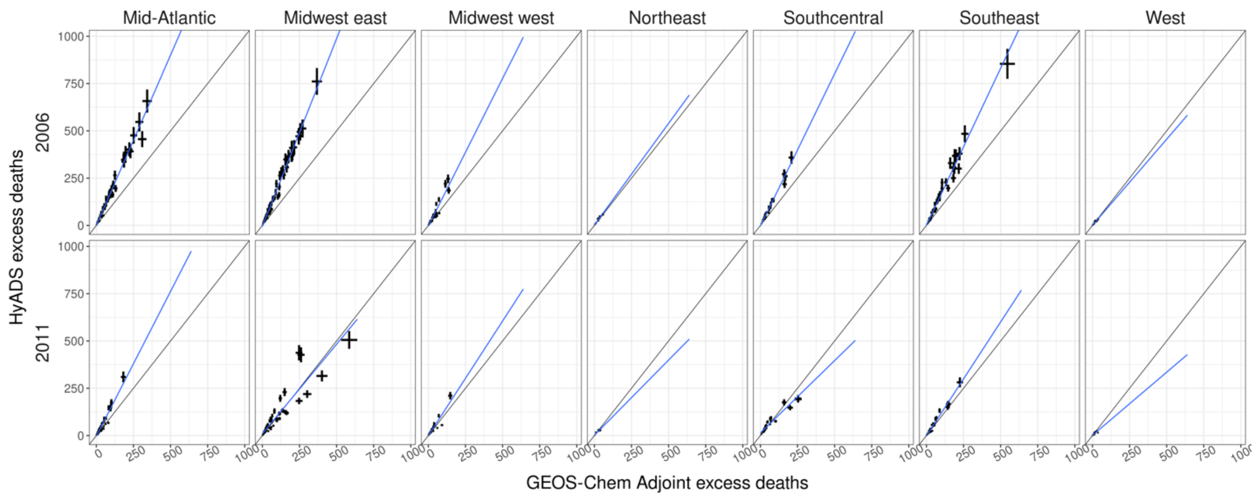

Fig. S9. Scatterplots of deaths associated with each coal EGU facility from the HyADS model against associated deaths derived using GEOS-Chem adjoint sensitivities. Each point represents a coal EGU facility located in each region, and the shading represents the linear regression standard error. Error bars in each point denote uncertainty from the CRF.

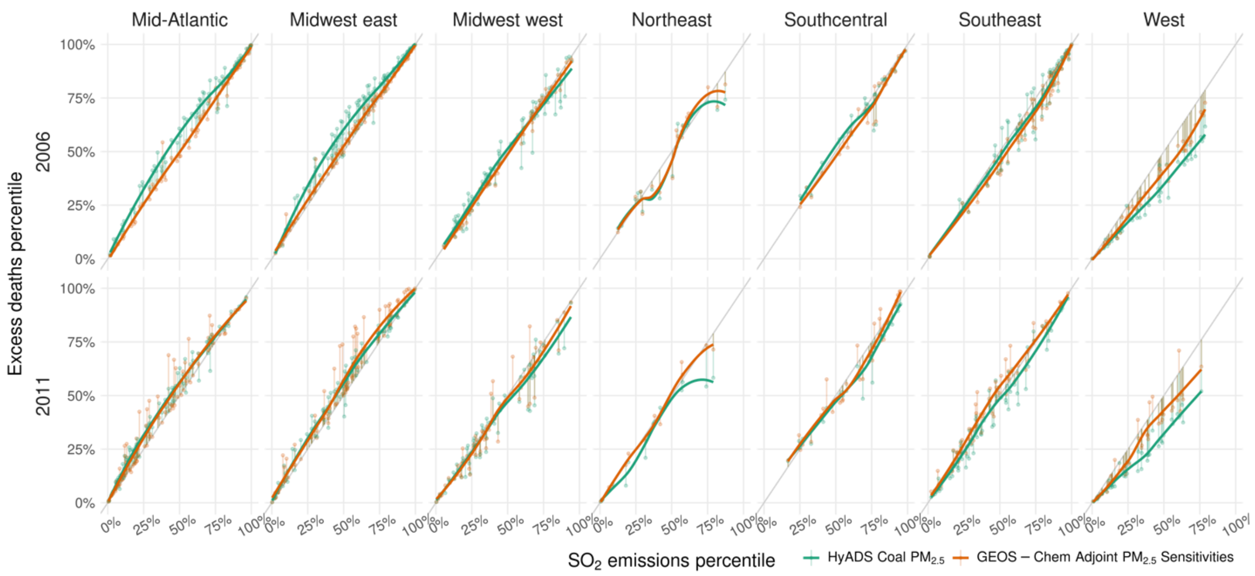

Fig. S10. National percentile rankings of each EGU facility's emissions (horizontal axis) and excess deaths (vertical axis) over the study period calculated using HyADS coal $PM_{2.5}$ and GEOS-Chem adjoint sensitivities. Points that fall above the 1:1 line represent facilities associated with relatively more deaths than expected based on their emissions rankings, and vice versa. Loess spline fits are presented to aid interpretability.

Table S1. Individual and ZIP code-level summary statistics for demographic data across all years (1999–2016) used in the stratified Poisson model. Individual characteristics reported in the Mean column represent the percent of all person-years (N = 655,889,210).

	Mean	Std. Dev.	Min	Max
**Individual characteristics**				
% Female	57			
% White	85			
% Black	8			
% Medicaid eligible	13			
Age	75	8	65	114
**Population characteristics**				
Mean BMI (kg m^{-2})	28	1	21	43
% ever smoker	47	8	0	100
% Hispanic population	9	16	0	100
% Black population	9	17	0	100
Median household income ($1000)	49	21	0	250
Median house value ($1000)	160	141	0	2,000
% below poverty level	11	10	0	100
% below high school education	29	19	0	100
Population density (person m^{-2})	1,553	5,108	0	153,868
% owner occupied housing	72	18	0	100
**Exposure and meteorology**				
Coal $PM_{2.5}$ (μg m^{-3})	0.96	1.23	0.0002	10.00
Summer temperature (K)	302	4	288	317
Winter temperature (K)	281	7	260	300
Summer relative humidity (%)	88	12	22	100
Winter relative humidity (%)	86	7	36	100

Table S2. Annual number of excess deaths attributable to coal $PM_{2.5}$ estimated using RRs for coal $PM_{2.5}$ from this study and RRs for total $PM_{2.5}$ from the literature. Numbers in parentheses denote 95% confidence interval. All excess deaths are estimated relative to zero coal $PM_{2.5}$. Analogous values are plotted in **Fig. 2**.

	Coal $PM_{2.5}$ (this study)	$PM_{2.5}$ (Wu et al., 2020) (4)	$PM_{2.5}$ (Krewski et al., 2009) (33)
1999	51,000 (46,000-56,000)	27,000 (24,000-29,000)	22,000 (14,000-31,000)
2000	41,000 (37,000-45,000)	22,000 (19,000-24,000)	18,000 (11,000-25,000)
2001	42,000 (38,000-46,000)	22,000 (20,000-24,000)	18,000 (12,000-25,000)
2002	38,000 (34,000-41,000)	20,000 (18,000-22,000)	17,000 (10,000-23,000)
2003	30,000 (27,000-33,000)	16,000 (14,000-17,000)	13,000 (8,400-18,000)
2004	29,000 (27,000-32,000)	15,000 (14,000-17,000)	13,000 (8,100-18,000)
2005	35,000 (32,000-38,000)	18,000 (17,000-20,000)	15,000 (9,700-21,000)
2006	35,000 (32,000-38,000)	18,000 (17,000-20,000)	15,000 (9,700-21,000)
2007	37,000 (34,000-41,000)	20,000 (18,000-21,000)	16,000 (10,000-23,000)
2008	27,000 (24,000-29,000)	14,000 (13,000-15,000)	12,000 (7,400-16,000)
2009	18,000 (16,000-20,000)	9,400 (8,500-10,000)	7,900 (5,000-11,000)
2010	14,000 (13,000-16,000)	7,600 (6,800-8,300)	6,300 (4,000-8,800)
2011	15,000 (13,000-16,000)	7,700 (6,900-8,500)	6,500 (4,100-8,900)
2012	9,700 (8,800-11,000)	5,100 (4,600-5,600)	4,300 (2,700-5,900)
2013	8,500 (7,700-9,300)	4,500 (4,000-4,900)	3,700 (2,400-5,100)
2014	8,000 (7,300-8,800)	4,200 (3,800-4,600)	3,500 (2,200-4,900)
2015	5,500 (4,900-6,000)	2,900 (2,600-3,100)	2,400 (1,500-3,300)
2016	3,500 (3,200-3,800)	1,800 (1,700-2,000)	1,500 (970-2,100)
2017	2,900 (2,700-3,200)	1,500 (1,400-1,700)	1,300 (810-1,800)
2018	2,800 (2,500-3,000)	1,400 (1,300-1,600)	1,200 (760-1,700)
2019	2,000 (1,800-2,100)	1,000 (930-1,100)	860 (540-1,200)
2020	1,600 (1,400-1,700)	830 (750-910)	690 (440-960)

Table S3. Coal $PM_{2.5}$ RR's per μg m^{-3} calculated using the Poisson model. The central estimates and confidence intervals for the "Residual $PM_{2.5}$" and "NO_2" models appear identical due to rounding.

	Central estimate (95% confidence interval)
Main model	1.0125 (1.0113, 1.0137)
Models trained on data from specific periods	
1999–2003	1.0129 (1.0115, 1.0142)
2004–2007	1.0126 (1.0111, 1.0141)
2008–2016	1.0285 (1.0260, 1.0310)
Models controlling for other pollutants (2000-2016)	
Total $PM_{2.5}$	1.0060 (1.0046, 1.0074)
Residual $PM_{2.5}$	1.0115 (1.0103, 1.0127)
NO_2	1.0115 (1.0103, 1.0127)
NO_2 and Residual $PM_{2.5}$	1.0109 (1.0097, 1.0122)

Table S4. Comparison of deaths associated with coal EGU facilities estimated using coal $PM_{2.5}$ with deaths derived using GEOS-Chem adjoint sensitivities. The RR applied in both models is from this work. Regions correspond to facility locations and associated deaths may occur in any region. Intercept and Slope terms correspond to a regression with HyADS coal $PM_{2.5}$ deaths as the dependent variable and GEOS-Chem adjoint sensitivity deaths as the independent variable. Normalized mean difference (NMD), normalized mean absolute difference (NMAD), and root mean square difference (RMSD) are facility-level comparisons of N observations of the HyADS deaths (P) vs. GEOS-Chem adjoint deaths (O). The metrics are defined as:

$$NMD = \frac{\sum_{i=1}^{N}(P_i - O_i)}{\sum_{i=1}^{N} O_i}$$

$$NMAD = \frac{\sum_{i=1}^{N}|P_i - O_i|}{\sum_{i=1}^{N} O_i}$$

$$RMSD = \sqrt{\frac{\sum_{i=1}^{N}(P_i - O_i)^2}{N}}$$

	Year	Intercept	Slope	R^2	NMD [%]	NMAD [%]	RMSD [deaths]
All facilities	2006	-4.8 ± 1	1.81 ± 0.01	0.97	71%	72%	57
	2011	4.4 ± 1	1.00 ± 0.02	0.87	15%	34%	21
Southeast	2006	0.1 ± 3	1.66 ± 0.03	0.98	66%	66%	61
	2011	-1.6 ± 1	1.20 ± 0.02	0.98	15%	23%	11
Southcentral	2006	-3.5 ± 3	1.61 ± 0.04	0.98	55%	55%	33
	2011	13.9 ± 3	0.76 ± 0.04	0.92	1%	25%	21
Midwest West	2006	-3.4 ± 2	1.56 ± 0.06	0.91	42%	46%	21
	2011	0.1 ± 2	1.21 ± 0.05	0.89	21%	34%	11
West	2006	0.6 ± 1	0.91 ± 0.04	0.94	-4%	15%	3
	2011	1.0 ± 1	0.67 ± 0.06	0.76	-20%	28%	4
Midwest East	2006	-5.3 ± 2	1.99 ± 0.02	0.99	90%	90%	73
	2011	9.5 ± 4	0.95 ± 0.04	0.85	12%	35%	35
Northeast	2006	2.1 ± 2	1.08 ± 0.06	0.96	17%	18%	4
	2011	1.8 ± 1	0.79 ± 0.06	0.89	-6%	25%	5
Mid-Atlantic	2006	-0.0 ± 2	1.80 ± 0.03	0.98	80%	80%	63
	2011	-2.9 ± 1	1.53 ± 0.04	0.94	39%	48%	19

Interactive data visualization tool
Link: https://cpieatgt.github.io/cpie/

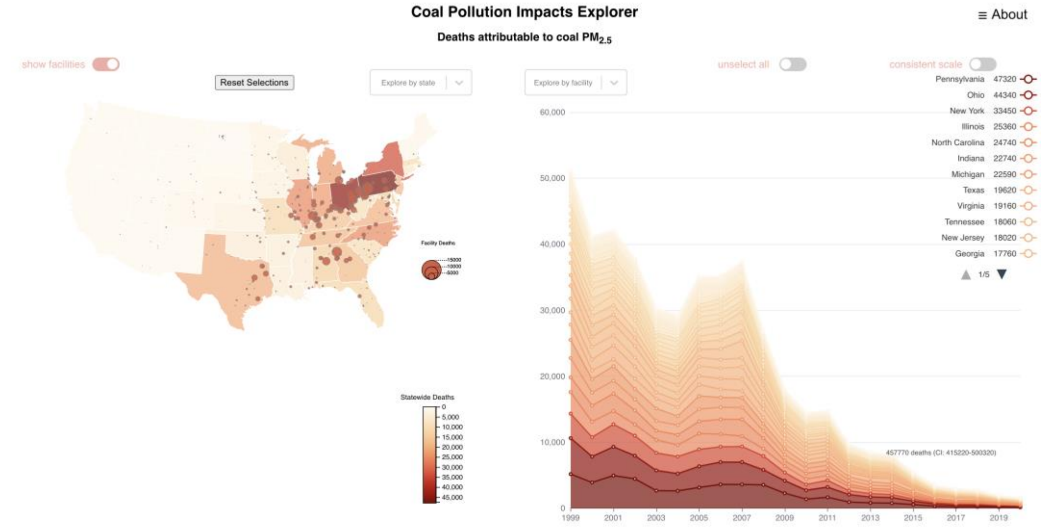

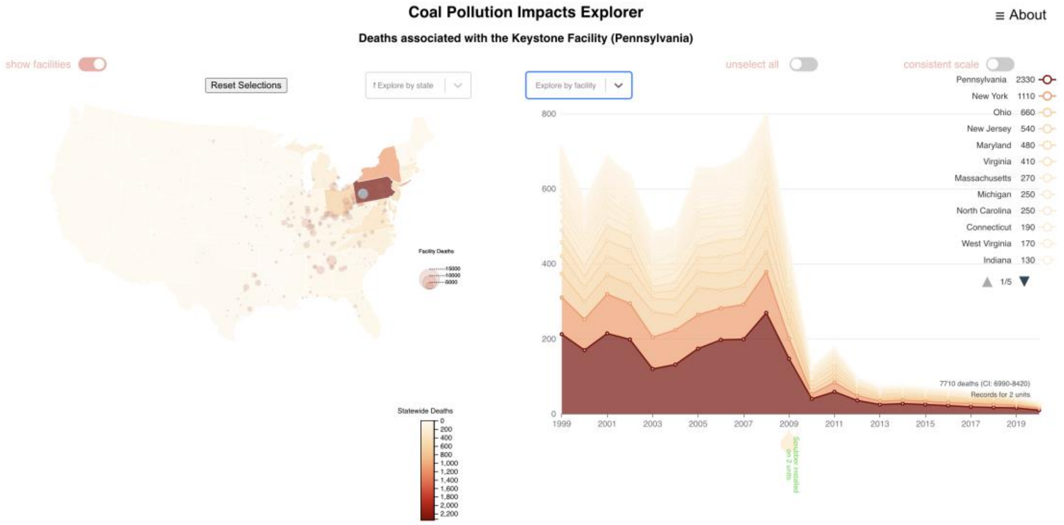

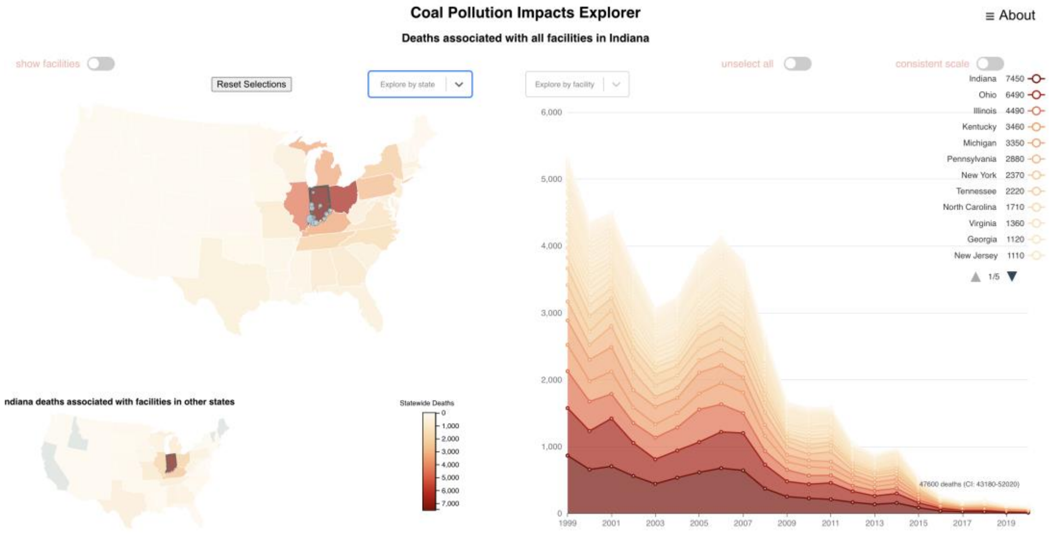

References and Notes

1. D. W. Dockery, C. A. Pope 3rd, X. Xu, J. D. Spengler, J. H. Ware, M. E. Fay, B. G. Ferris Jr., F. E. Speizer, An association between air pollution and mortality in six U.S. cities. *N. Engl. J. Med.* **329**, 1753–1759 (1993). doi:10.1056/NEJM199312093292401 Medline

2. F. Laden, J. Schwartz, F. E. Speizer, D. W. Dockery, Reduction in fine particulate air pollution and mortality: Extended follow-up of the Harvard Six Cities study. *Am. J. Respir. Crit. Care Med.* **173**, 667–672 (2006). doi:10.1164/rccm.200503-443OC Medline

3. Q. Di, Y. Wang, A. Zanobetti, Y. Wang, P. Koutrakis, C. Choirat, F. Dominici, J. D. Schwartz, Air pollution and mortality in the Medicare population. *N. Engl. J. Med.* **376**, 2513–2522 (2017). doi:10.1056/NEJMoa1702747 Medline

4. X. Wu, D. Braun, J. Schwartz, M. A. Kioumourtzoglou, F. Dominici, Evaluating the impact of long-term exposure to fine particulate matter on mortality among the elderly. *Sci. Adv.* **6**, eaba5692 (2020). doi:10.1126/sciadv.aba5692 Medline

5. J. M. Godowitch, G. Pouliot, S. Trivikrama Rao, Assessing multi-year changes in modeled and observed urban NOX concentrations from a dynamic model evaluation perspective. *Atmos. Environ.* **44**, 2894–2901 (2010). doi:10.1016/j.atmosenv.2010.04.040

6. J. A. de Gouw, D. D. Parrish, G. J. Frost, M. Trainer, Reduced emissions of CO_2, NO_x, and SO_2 from U.S. power plants due to the switch from coal to natural gas with combined cycle technology. *Earths Futur.* **2**, 75–82 (2014). doi:10.1002/2013EF000196

7. L. Xu, H. Guo, C. M. Boyd, M. Klein, A. Bougiatioti, K. M. Cerully, J. R. Hite, G. Isaacman-VanWertz, N. M. Kreisberg, C. Knote, K. Olson, A. Koss, A. H. Goldstein, S. V. Hering, J. de Gouw, K. Baumann, S.-H. Lee, A. Nenes, R. J. Weber, N. L. Ng, Effects of anthropogenic emissions on aerosol formation from isoprene and monoterpenes in the southeastern United States. *Proc. Natl. Acad. Sci. U.S.A.* **112**, 37–42 (2015). doi:10.1073/pnas.1417609112 Medline

8. International Energy Agency, “Coal 2022: Analysis and forecast to 2025” (IEA, 2022); https://www.iea.org/reports/coal-2022.

9. S. Meredith, “Russia is squeezing Europe’s gas supplies, sparking a bitter and reluctant return to coal,” *CNBC*, 21 June 2022; https://www.cnbc.com/2022/06/21/ukraine-war-europe-turns-to-coal-as-russia-squeezes-gas-supplies.html.

10. M. Eddy, “Germany will fire up coal plants again in an effort to save natural gas,” *The New York Times*, 19 June 2022; https://www.nytimes.com/2022/06/19/world/europe/germany-russia-gas.html.

11. I. C. Dedoussi, F. Allroggen, R. Flanagan, T. Hansen, B. Taylor, S. R. H. Barrett, J. K. Boyce, The co-pollutant cost of carbon emissions: An analysis of the US electric power generation sector. *Environ. Res. Lett.* **14**, 094003 (2019). doi:10.1088/1748-9326/ab34e3

12. US Environmental Protection Agency, “New source performance standards for greenhouse gas emissions from new, modified, and reconstructed fossil fuel-fired electric generating units; emission guidelines for greenhouse gas emissions from existing fossil fuel-fired electric generating units; and repeal of the Affordable Clean Energy Rule” (EPA, 2023);

https://www.epa.gov/system/files/documents/2023-05/FRL-8536-02-OAR%20111EGU%20NPRM%2020230504_Admin.pdf.

13. I. C. Dedoussi, S. D. Eastham, E. Monier, S. R. H. Barrett, Premature mortality related to United States cross-state air pollution. *Nature* **578**, 261–265 (2020). doi:10.1038/s41586-020-1983-8 Medline

14. M. P. S. Thind, C. W. Tessum, I. L. Azevedo, J. D. Marshall, Fine particulate air pollution from electricity generation in the US: Health impacts by race, income, and geography. *Environ. Sci. Technol.* **53**, 14010–14019 (2019). doi:10.1021/acs.est.9b02527 Medline

15. J. J. Buonocore, X. Dong, J. D. Spengler, J. S. Fu, J. I. Levy, Using the Community Multiscale Air Quality (CMAQ) model to estimate public health impacts of $PM_{2.5}$ from individual power plants. *Environ. Int.* **68**, 200–208 (2014). doi:10.1016/j.envint.2014.03.031 Medline

16. F. Caiazzo, A. Ashok, I. A. Waitz, S. H. L. Yim, S. R. H. Barrett, Air pollution and early deaths in the United States. Part I: Quantifying the impact of major sectors in 2005. *Atmos. Environ.* **79**, 198–208 (2013). doi:10.1016/j.atmosenv.2013.05.081

17. I. C. Dedoussi, S. R. H. Barrett, Air pollution and early deaths in the United States. Part II: Attribution of PM2.5 exposure to emissions species, time, location and sector. *Atmos. Environ.* **99**, 610–617 (2014). doi:10.1016/j.atmosenv.2014.10.033

18. J. Lelieveld, K. Klingmüller, A. Pozzer, R. T. Burnett, A. Haines, V. Ramanathan, Effects of fossil fuel and total anthropogenic emission removal on public health and climate. *Proc. Natl. Acad. Sci. U.S.A.* **116**, 7192–7197 (2019). doi:10.1073/pnas.1819989116 Medline

19. P. K. Hopke, D. P. Croft, W. Zhang, S. Lin, M. Masiol, S. Squizzato, S. W. Thurston, E. van Wijngaarden, M. J. Utell, D. Q. Rich, Changes in the hospitalization and ED visit rates for respiratory diseases associated with source-specific $PM_{2.5}$ in New York State from 2005 to 2016. *Environ. Res.* **181**, 108912 (2020). doi:10.1016/j.envres.2019.108912 Medline

20. T. To, J. Zhu, E. Terebessy, K. Zhang, I. Fong, L. Pinault, M. Jerrett, A. Robichaud, R. Ménard, A. van Donkelaar, R. V. Martin, P. Hystad, J. R. Brook, S. Dell, D. Stieb, Does exposure to air pollution increase the risk of acute care in young children with asthma? An Ontario, Canada study. *Environ. Res.* **199**, 111302 (2021). doi:10.1016/j.envres.2021.111302 Medline

21. B. Ostro, J. Hu, D. Goldberg, P. Reynolds, A. Hertz, L. Bernstein, M. J. Kleeman, Associations of mortality with long-term exposures to fine and ultrafine particles, species and sources: Results from the California Teachers Study Cohort. *Environ. Health Perspect.* **123**, 549–556 (2015). doi:10.1289/ehp.1408565 Medline

22. Y. Wang, S. Xiao, Y. Zhang, H. Chang, R. V. Martin, A. Van Donkelaar, A. Gaskins, Y. Liu, P. Liu, L. Shi, Long-term exposure to $PM_{2.5}$ major components and mortality in the southeastern United States. *Environ. Int.* **158**, 106969 (2022). doi:10.1016/j.envint.2021.106969 Medline

23. L. R. F. Henneman, C. Choirat, A. C. M. Zigler, Accountability assessment of health improvements in the United States associated with reduced coal emissions between 2005

and 2012. *Epidemiology* **30**, 477–485 (2019). doi:10.1097/EDE.0000000000001024 Medline

24. S. Weichenthal, E. Lavigne, A. Traub, D. Umbrio, H. You, K. Pollitt, T. Shin, R. Kulka, D. M. Stieb, J. Korsiak, B. Jessiman, J. R. Brook, M. Hatzopoulou, G. Evans, R. T. Burnett, Association of sulfur, transition metals, and the oxidative potential of outdoor PM2.5 with acute cardiovascular events: A case-crossover study of Canadian adults. *Environ. Health Perspect.* **129**, 107005 (2021). doi:10.1289/EHP9449 Medline

25. G. D. Thurston, R. T. Burnett, M. C. Turner, Y. Shi, D. Krewski, R. Lall, K. Ito, M. Jerrett, S. M. Gapstur, W. R. Diver, C. A. Pope, Ischemic heart disease mortality and long-term exposure to source-related components of U.S. fine particle air pollution. *Environ. Health Perspect.* **124**, 785–794 (2016). doi:10.1289/ehp.1509777 Medline

26. Z. A. Pond, C. S. Hernandez, P. J. Adams, S. N. Pandis, G. R. Garcia, A. L. Robinson, J. D. Marshall, R. Burnett, K. Skyllakou, P. Garcia Rivera, E. Karnezi, C. J. Coleman, C. A. Pope 3rd, Cardiopulmonary mortality and fine particulate air pollution by species and source in a national U.S. cohort. *Environ. Sci. Technol.* **56**, 7214–7223 (2022). doi:10.1021/acs.est.1c04176 Medline

27. D. Q. Rich, W. Zhang, S. Lin, S. Squizzato, S. W. Thurston, E. van Wijngaarden, D. Croft, M. Masiol, P. K. Hopke, Triggering of cardiovascular hospital admissions by source specific fine particle concentrations in urban centers of New York State. *Environ. Int.* **126**, 387–394 (2019). doi:10.1016/j.envint.2019.02.018 Medline

28. Centers for Medicare & Medicaid; https://www.cms.gov/.

29. J. A. Casey, J. G. Su, L. R. F. Henneman, C. Zigler, A. M. Neophytou, R. Catalano, R. Gondalia, Y.-T. Chen, L. Kaye, S. S. Moyer, V. Combs, G. Simrall, T. Smith, J. Sublett, M. A. Barrett, Improved asthma outcomes observed in the vicinity of coal power plant retirement, retrofit, and conversion to natural gas. *Nat. Energy* **5**, 398–408 (2020). doi:10.1038/s41560-020-0600-2 Medline

30. L. R. F. Henneman, I. C. Dedoussi, J. A. Casey, C. Choirat, S. R. H. Barrett, C. M. Zigler, Comparisons of simple and complex methods for quantifying exposure to individual point source air pollution emissions. *J. Expo. Sci. Environ. Epidemiol.* **31**, 654–663 (2021). doi:10.1038/s41370-020-0219-1 Medline

31. L. R. F. Henneman, C. Choirat, C. Ivey, K. Cummiskey, C. M. Zigler, Characterizing population exposure to coal emissions sources in the United States using the HyADS model. *Atmos. Environ.* **203**, 271–280 (2019). doi:10.1016/j.atmosenv.2019.01.043 Medline

32. L. R. Henneman, L. J. Mickley, C. M. Zigler, Air pollution accountability of energy transitions: The relative importance of point source emissions and wind fields in exposure changes. *Environ. Res. Lett.* **14**, 115003 (2019). doi:10.1088/1748-9326/ab4861 Medline

33. D. Krewski, M. Jerrett, R. T. Burnett, R. Ma, E. Hughes, Y. Shi, M. C. Turner, C. A. Pope 3rd, G. Thurston, E. E. Calle, M. J. Thun, B. Beckerman, P. DeLuca, N. Finkelstein, K. Ito, D. K. Moore, K. B. Newbold, T. Ramsay, Z. Ross, H. Shin, B. Tempalski, Extended follow-up and spatial analysis of the American Cancer Society study linking particulate

air pollution and mortality. *Res. Rep. Health Eff. Inst.* **140**, 5–114, discussion 115–136 (2009). Medline

34. T. J. VanderWeele, P. Ding, Sensitivity analysis in observational research: Introducing the E-value. *Ann. Intern. Med.* **167**, 268–274 (2017). doi:10.7326/M16-2607 Medline

35. M. B. Mathur, P. Ding, C. A. Riddell, T. J. VanderWeele, Web site and R package for computing E-values. *Epidemiology* **29**, e45–e47 (2018). doi:10.1097/EDE.0000000000000864 Medline

36. K. P. Josey, P. deSouza, X. Wu, D. Braun, R. Nethery, Estimating a causal exposure response function with a continuous error-prone exposure: A study of fine particulate matter and all-cause mortality. *J. Agric. Biol. Environ. Stat.* **28**, 20–41 (2023). doi:10.1007/s13253-022-00508-z Medline

37. C. W. Tessum, J. S. Apte, A. L. Goodkind, N. Z. Muller, K. A. Mullins, D. A. Paolella, S. Polasky, N. P. Springer, S. K. Thakrar, J. D. Marshall, J. D. Hill, Inequity in consumption of goods and services adds to racial-ethnic disparities in air pollution exposure. *Proc. Natl. Acad. Sci. U.S.A.* **116**, 6001–6006 (2019). doi:10.1073/pnas.1818859116 Medline

38. US Environmental Protection Agency, "Air pollutant emissions trends data: State average annual emissions trend" (EPA, 2018); https://www.epa.gov/air-emissions-inventories/air-pollutant-emissions-trends-data.

39. E. A. Gilmore, J. Heo, N. Z. Muller, C. W. Tessum, J. D. Hill, J. D. Marshall, P. J. Adams, An inter-comparison of the social costs of air quality from reduced-complexity models. *Environ. Res. Lett.* **14**, 074016 (2019). doi:10.1088/1748-9326/ab1ab5

40. S. L. Penn, S. Arunachalam, M. Woody, W. Heiger-Bernays, Y. Tripodis, J. I. Levy, Estimating state-specific contributions to PM2.5- and O3-related health burden from residential combustion and electricity generating unit emissions in the United States. *Environ. Health Perspect.* **125**, 324–332 (2017). doi:10.1289/EHP550 Medline

41. N. Fann, C. M. Fulcher, K. Baker, The recent and future health burden of air pollution apportioned across U.S. sectors. *Environ. Sci. Technol.* **47**, 3580–3589 (2013). doi:10.1021/es304831q Medline

42. P. K. Hopke, Review of receptor modeling methods for source apportionment. *J. Air Waste Manag. Assoc.* **66**, 237–259 (2016). doi:10.1080/10962247.2016.1140693 Medline

43. S. L. Napelenok, D. S. Cohan, Y. Hu, A. G. Russell, Decoupled direct 3D sensitivity analysis for particulate matter (DDM-3D/PM). *Atmos. Environ.* **40**, 6112–6121 (2006). doi:10.1016/j.atmosenv.2006.05.039

44. R. H. F. Kwok, K. R. Baker, S. L. Napelenok, G. S. Tonnesen, Photochemical grid model implementation and application of VOC, NO_x, and O_3 source apportionment. *Geosci. Model Dev.* **8**, 99–114 (2015). doi:10.5194/gmd-8-99-2015

45. Clean Air Science Advisory Committee, "CASAC review of the EPA's policy assessment for the reconsideration of the National Ambient Air Quality Standards for Particulate Matter" (EPA, 2022), report EPA-CASAC-22-002; https://casac.epa.gov/ords/sab/f?p=113:12:1342972375271:::12.

46. US Environmental Protection Agency, "Clean air markets program data" (EPA, 2016); https://campd.epa.gov/.

47. Online data exploration tool "Coal Pollution Impacts Explorer" for: S. Jin, L. Henneman, C. Choirat, I. Dedoussi, F. Dominici, C. Zigler, J. Roberts, Mortality risk from United States coal electricity generation, GitHub (2023); https://cpieatgt.github.io/cpie/.

48. Code for: S. Jin, L. Henneman, C. Choirat, I. Dedoussi, F. Dominici, C. Zigler, J. Roberts, Mortality risk from United States coal electricity generation, GitHub (2023); https://github.com/NSAPH-Projects/coal_pm25-mortality-medicare.

49. Coal PM2.5 source impacts and coal EGU information for: S. Jin, L. Henneman, C. Choirat, I. Dedoussi, F. Dominici, C. Zigler, J. Roberts, Mortality risk from United States coal electricity generation, (OSF, 2021); https://doi.org/10.17605/OSF.IO/8GDAU.

50. B. Sabath, "National Causal Analysis Health Outcomes: SAS code to create analytic dataset, GitHub (2021); https://github.com/NSAPH/National-Causal-Analysis/tree/master/HealthOutcomes.

51. M. Daouda, L. Henneman, M.-A. Kioumourtzoglou, A. Gemmill, C. Zigler, J. Casey, Association between county-level coal-fired power plant pollution and racial disparities in preterm births from 2000 to 2018. *Environ. Res. Lett.* **16**, 034055 (2021). doi:10.1088/1748-9326/abe4f7 Medline

52. R. R. Draxler, G. D. Hess, An overview of the HYSPLIT_4 modelling system for trajectories, dispersion, and deposition. *Aust. Meteorol. Mag.* **47**, 295–308 (1998).

53. US Environmental Protection Agency, "Monitoring insights: Relative accuracy" (EPA, 2022); https://www.epa.gov/system/files/documents/2022-05/Monitoring%20Insights-%20Relative%20Accuracy.pdf.

54. C. Ivey, H. Holmes, G. Shi, S. Balachandran, Y. Hu, A. G. Russell, Development of $PM_{2.5}$ Source profiles using a hybrid chemical transport-receptor modeling approach. *Environ. Sci. Technol.* **51**, 13788–13796 (2017). doi:10.1021/acs.est.7b03781 Medline

55. L. R. F. Henneman, M. M. Rasel, C. Choirat, S. C. Anenberg, C. Zigler, Inequitable exposures to U.S. coal power plant-related $PM_{2.5}$: 22 years and counting. *Environ. Health Perspect.* **131**, 37005 (2023). doi:10.1289/EHP11605 Medline

56. D. B. Gingerich, Y. Zhao, M. S. Mauter, Environmentally significant shifts in trace element emissions from coal plants complying with the 1990 Clean Air Act Amendments. *Energy Policy* **132**, 1206–1215 (2019). doi:10.1016/j.enpol.2019.07.003

57. A. Dasgupta, *Asymptotic Theory of Statistics and Probability* (Springer, 2008); http://link.springer.com/10.1007/978-0-387-75971-5.

58. M. M. Nasari, M. Szyszkowicz, H. Chen, D. Crouse, M. C. Turner, M. Jerrett, C. A. Pope 3rd, B. Hubbell, N. Fann, A. Cohen, S. M. Gapstur, W. R. Diver, D. Stieb, M. H. Forouzanfar, S.-Y. Kim, C. Olives, D. Krewski, R. T. Burnett, A class of non-linear exposure-response models suitable for health impact assessment applicable to large cohort studies of ambient air pollution. *Air Qual. Atmos. Health* **9**, 961–972 (2016). doi:10.1007/s11869-016-0398-z Medline

59. J. D. Sacks, J. M. Lloyd, Y. Zhu, J. Anderton, C. J. Jang, B. Hubbell, N. Fann, The Environmental Benefits Mapping and Analysis Program - Community Edition (BenMAP-CE): A tool to estimate the health and economic benefits of reducing air pollution. *Environ. Model. Softw.* **104**, 118–129 (2018). doi:10.1016/j.envsoft.2018.02.009 Medline

60. R. T. Burnett, C. A. Pope 3rd, M. Ezzati, C. Olives, S. S. Lim, S. Mehta, H. H. Shin, G. Singh, B. Hubbell, M. Brauer, H. R. Anderson, K. R. Smith, J. R. Balmes, N. G. Bruce, H. Kan, F. Laden, A. Prüss-Ustün, M. C. Turner, S. M. Gapstur, W. R. Diver, A. Cohen, An integrated risk function for estimating the global burden of disease attributable to ambient fine particulate matter exposure. *Environ. Health Perspect.* **122**, 397–403 (2014). doi:10.1289/ehp.1307049 Medline

61. US Environmental Protection Agency, "2017 National Emissions Inventory: January 2021 Updated Release, Technical Support Document" (EPA, 2021), Report EPA-454-21-001; https://www.epa.gov/sites/default/files/2021-02/documents/nei2017_tsd_full_jan2021.pdf.

62. Q. Di, H. Amini, L. Shi, I. Kloog, R. Silvern, J. Kelly, M. B. Sabath, C. Choirat, P. Koutrakis, A. Lyapustin, Y. Wang, L. J. Mickley, J. Schwartz, Assessing NO_2 concentration and model uncertainty with high spatiotemporal resolution across the contiguous United States using ensemble model averaging. *Environ. Sci. Technol.* **54**, 1372–1384 (2020). doi:10.1021/acs.est.9b03358 Medline

63. J. Holt, N. E. Selin, S. Solomon, Changes in inorganic fine particulate matter sensitivities to precursors due to large-scale US emissions reductions. *Environ. Sci. Technol.* **49**, 4834–4841 (2015). doi:10.1021/acs.est.5b00008 Medline

64. P. A. Solomon, D. Crumpler, J. B. Flanagan, R. K. M. Jayanty, E. E. Rickman, C. E. McDade, U.S. national PM2.5 Chemical Speciation Monitoring Networks-CSN and IMPROVE: Description of networks. *J. Air Waste Manag. Assoc.* **64**, 1410–1438 (2014). doi:10.1080/10962247.2014.956904 Medline
